# Identification of PUFA interaction sites on the cardiac potassium channel KCNQ1

**DOI:** 10.1085/jgp.202012850

**Published:** 2021-05-03

**Authors:** Samira Yazdi, Johan Nikesjö, Williams Miranda, Valentina Corradi, D. Peter Tieleman, Sergei Yu. Noskov, H. Peter Larsson, Sara I. Liin

**Affiliations:** 1 Department of Biomedical and Clinical Sciences, Linköping University, Linköping, Sweden; 2 Centre for Molecular Simulations, Department of Biological Sciences, University of Calgary, Calgary, AB, Canada; 3 Department of Physiology and Biophysics, University of Miami, Miami, FL

## Abstract

Polyunsaturated fatty acids (PUFAs), but not saturated fatty acids, modulate ion channels such as the cardiac KCNQ1 channel, although the mechanism is not completely understood. Using both simulations and experiments, we find that PUFAs interact directly with the KCNQ1 channel via two different binding sites: one at the voltage sensor and one at the pore. These two amphiphilic binding pockets stabilize the negatively charged PUFA head group by electrostatic interactions with R218, R221, and K316, while the hydrophobic PUFA tail is selectively stabilized by cassettes of hydrophobic residues. The rigid saturated tail of stearic acid prevents close contacts with KCNQ1. By contrast, the mobile tail of PUFA linoleic acid can be accommodated in the crevice of the hydrophobic cassette, a defining feature of PUFA selectivity in KCNQ1. In addition, we identify Y268 as a critical PUFA anchor point underlying fatty acid selectivity. Combined, this study provides molecular models of direct interactions between PUFAs and KCNQ1 and identifies selectivity mechanisms. Long term, this understanding may open new avenues for drug development based on PUFA mechanisms.

## Teaser

The cardiac KCNQ1 channel is a promising anti-arrhythmic target. Yazdi et al. report on how PUFAs interact with two binding sites in KCNQ1 to trigger channel activation. These findings further our mechanistic understanding of how to modulate KCNQ1 activity.

## Introduction

Polyunsaturated fatty acids (PUFAs) affect many different ion channels, such as voltage-gated K^+^, Na^+^, and Ca^2+^ channels, as well as ryanodine receptors (Xiao et al., 2001; Hamilton et al., 2003; Oliver et al., 2004; Xiao et al., 2005; Ottosson et al., 2014; Farag et al., 2016; Tian et al., 2016; Liin et al., 2018; Bohannon et al., 2020). However, the molecular mechanisms behind the effects of PUFAs are not completely understood. The identity of PUFAs’ specific binding pockets remains uncharacterized for many ion channels; therefore, the molecular mechanisms of PUFA efficacy are still poorly understood. Atomistic understanding of PUFA binding sites and mechanisms of action on ion channels would further our ability to design PUFAs that modulate ion channel function and aid in the development of drugs based on PUFA mechanisms.

We previously showed that PUFAs act as activators of KCNQ1 (Liin et al., 2015), which is expressed, for instance, in cardiomyocytes (forming the KCNQ1/KCNE1 channel) and smooth muscle cells (Barrese et al., 2018). KCNQ1 forms a tetrameric, voltage-gated potassium channel with six transmembrane segments (S1–S6) in each subunit (Sun and MacKinnon, 2017). Recently, a cryo-EM structure of the *Xenopus laevis* KCNQ1 (xKCNQ1) channel was published (Sun and MacKinnon, 2017). Helices S1–S4 in each subunit form a voltage-sensing domain with positively charged arginine residues in S4 acting as the voltage sensor. S5–S6 from all four subunits together form the centrally located pore domain with the potassium selectivity filter and activation gate. Outward movement of the positively charged S4 in response to depolarization triggers opening of the activation gate and allows for the outward flux of potassium (Osteen et al., 2012; Barro-Soria et al., 2014; Zaydman et al., 2014; Hou et al., 2017; Westhoff et al., 2019).

**Figure 1.**
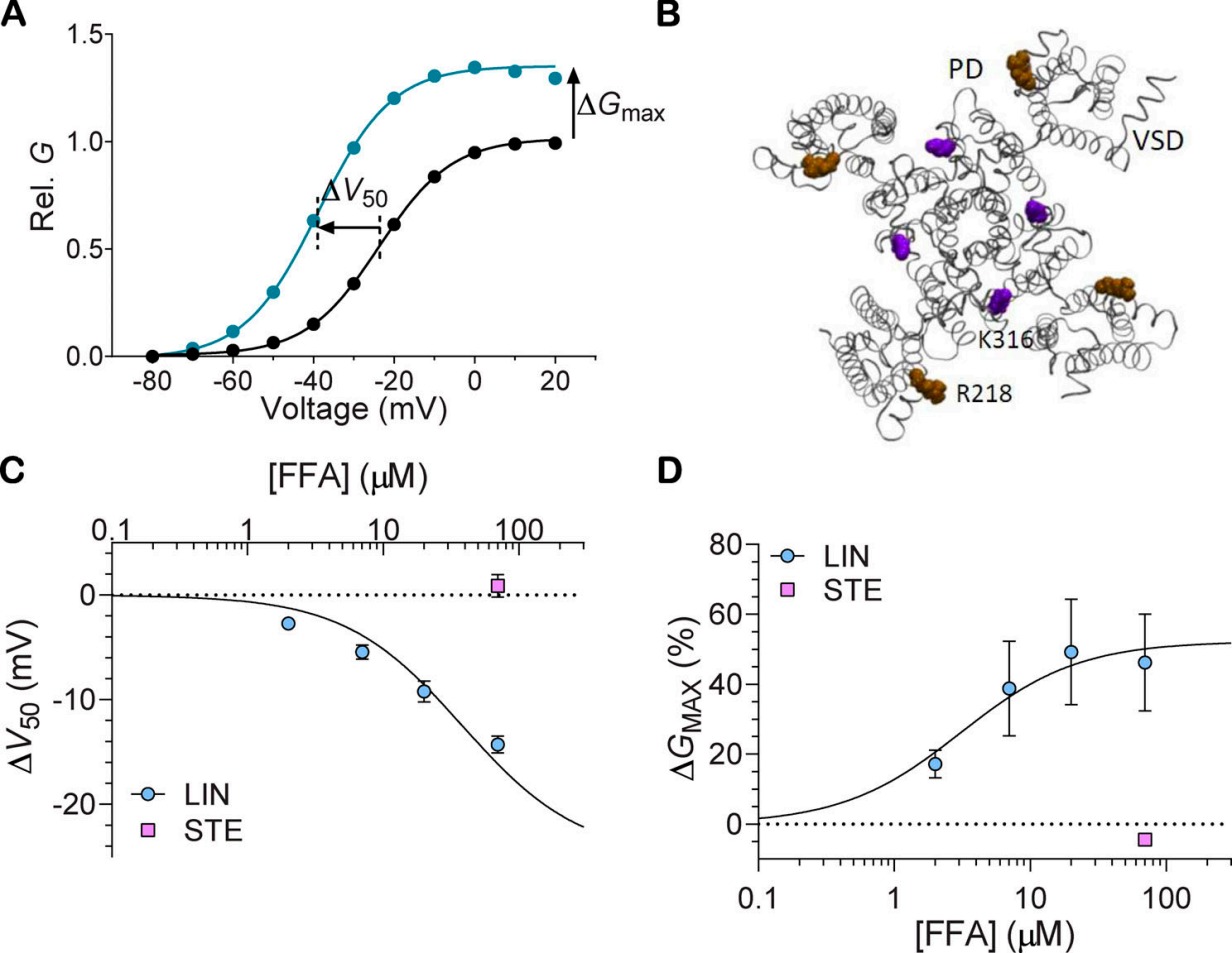
**Effect of PUFA on KCNQ1 channel.** **(A)** Representative example of the effect of PUFA on the KCNQ1 channel, showing a shift in the voltage dependence for channel opening toward negative voltages (Δ*V*_50_) and an increase in the maximal conductance (Δ*G*_max_). Curves represent Boltzmann fits to control (black line) and 20 µM of the PUFA LIN (blue). **(B)** Top view of the xKCNQ1 structure with the pore domain in gray and the voltage-sensing domain in black. xKCNQ1 residues R218 and K316 (corresponding to R228 and K326, respectively, in hKCNQ1) are highlighted in brown and purple, respectively. Previous studies identifying these residues as important for PUFA effects used N-AT and DHA-Gly (Liin et al., 2015; Liin et al., 2018). **(C and D)** Effect of LIN or STE at indicated free FA (FFA) concentrations on *V*_50_ (C) or *G*_max_ (D) of WT hKCNQ1. Data shown as mean ± SEM; *n* = 5–8 per data point. Concentration-response curves for LIN were fitted using Eq. 2 with the Hill coefficient constrained to either 1 or −1 (see Materials and methods for details). Δ*V*_50,max_ was constrained to −25 mV to make the fit more robust. Δ*V*_50,max_ denotes maximal effect in Δ*V*_50_; Rel., relative.

Experiments combining electrophysiology and molecular biology provided evidence that PUFAs affect both the voltage sensor and the pore of KCNQ1 channels (Liin et al., 2016; Liin et al., 2018). PUFAs shift the voltage dependence of channel opening (detected as a shift in the voltage at which the conductance is half the maximal conductance [Δ*V*_50_] in Fig. 1 A). The outermost S4 arginine, R218 in xKCNQ1 (which corresponds to R228 in human KCNQ1; hKCNQ1), is important for this effect (Fig. 1 B; Liin et al., 2015; Liin et al., 2018). PUFAs also increase the maximum conductance of KCNQ1 channels (Δ*G*_max_ in Fig. 1 A). A conserved positively charged S6 residue, K316 in xKCNQ1 (which corresponds to K326 in hKCNQ1), is important for this increase (Fig. 1 B; Liin et al., 2018). The two effects of PUFAs on KCNQ1 channels are independent of each other (Liin et al., 2018). However, our previous studies have not allowed us to determine if there is one PUFA binding site on KCNQ1, from which one PUFA molecule exerts the two effects, or if there are two PUFA binding sites on KCNQ1: one close to the voltage sensor and one close to the pore. Moreover, molecular understanding of why PUFAs such as linoleic acid (LIN), but not monounsaturated or saturated fatty acids (FAs; such as the saturated stearic acid [STE]), activate KCNQ1 channels (Fig. 1, C and D; Liin et al., 2015) and how the PUFA tail interacts with KCNQ1 channels are still unclear. Here, we conducted computer simulations combined with electrophysiology experiments to address these open questions.

## Materials and methods

### Structure preparation

The 3-D structure used for the transmembrane domain of KCNQ1 channel (residues 94–348) was built using the recently published structure of xKCNQ1 channel solved by cryo-EM at 3.7 Å (Sun and MacKinnon, 2017). Throughout the Results section, xKCNQ1 numbering of residues is used if not stated otherwise. In this structure of KCNQ1, S4 is in an activated “up” position, whereas the pore is closed. Although PUFA interaction with the pore domain could potentially be different in closed and open channels, we anticipate any such differences to be minor for the following reasons. Previous work on K_V_ channels show that the main structural differences between a closed and open pore is at the internal gate of the pore, which is in the intracellular half of the channel (Bavro et al., 2012; Jensen et al., 2012; Lenaeus et al., 2017; Sun and MacKinnon, 2017; Kuenze et al., 2019). In contrast, limited structural differences between closed and open pores are seen at the lipid-facing surface of the extracellular half of the pore (Bavro et al., 2012; Jensen et al., 2012; Lenaeus et al., 2017; Sun and MacKinnon, 2017; Kuenze et al., 2019), which is the part of the channel with which PUFA interacts. Moreover, previous experiments suggest that PUFA interaction with the pore domain increases the maximum conductance of KCNQ1 by affecting the selectivity filter (Liin et al., 2018), and the selectivity filter, which is located in the extracellular half of the channel, is not expected to change much between closed and open states (Cordero-Morales et al., 2006; Bavro et al., 2012; Jensen et al., 2012; Lenaeus et al., 2017).

**Figure 2.**
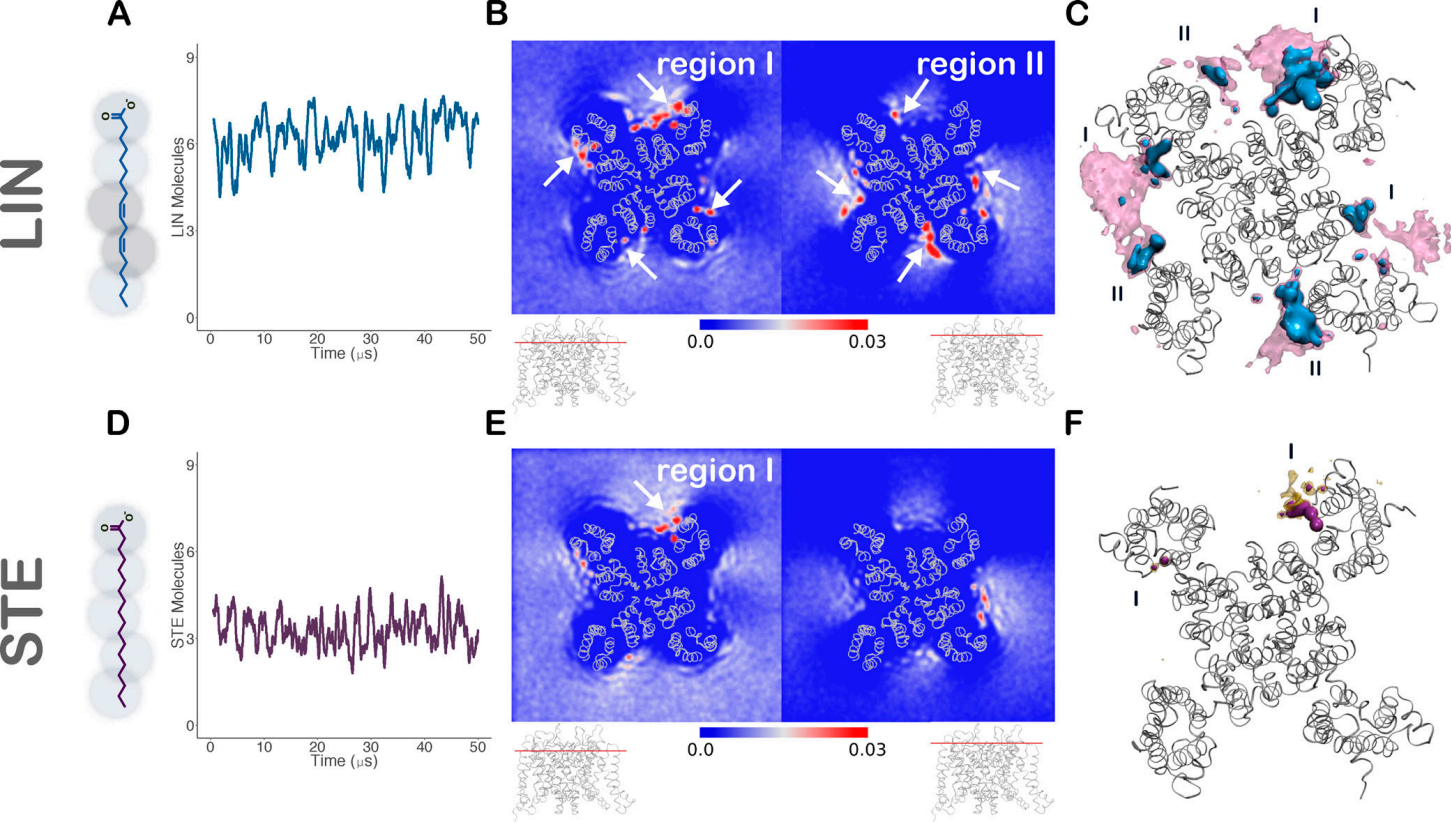
**LIN and STE distribution around KCNQ1.** **(A** **and D)** Left: 2-D structure of LIN (A), an 18-carbon chain with two cis double bonds, and STE (D), a fully saturated 18-carbon chain, overlaid with the Martini CG models of LIN and STE, respectively. Right: Running average over 500 ns of the number of LIN (A) and STE (D) molecules within 6 Å of the channel in the upper bilayer. **(B and E)** 2-D occupancy maps of LIN (B) and STE (E) head groups around KCNQ1 shown as mean occupancies during the interval of 35–50 µs of the simulation. 0.03 in color scale corresponds to a 3% occupancy (i.e., LIN head group present in 3% of all frames in B). Top-down view of the channel in ribbon representation to clarify LIN (B) and STE (E) enrichment regions. Densities at different layers along the z axis (red line in channel side view below the density maps). Arrows highlight LIN or STE enrichment at regions I and II.** (C and F) **3-D contour map of the occupancy of LIN (C) and STE (F) around KCNQ1. Threshold values are set at 15% (mauve or yellow) and 20% (blue or purple) of the total time (a LIN molecule is present in C at least 15% and 20% of the frames, respectively). LIN enrichment regions I and II are indicated in C.

CHARMM-GUI Martini Bilayer Maker (Jo et al., 2008) was used to prepare the system containing the protein embedded in a pure phosphatidylcholine bilayer solvated in 0.1 M KCl aqueous solution using CHARMM36 force field and TIP3P water model (Jorgensen et al., 1983; Noskov et al., 2004; Noskov and Roux, 2008). The system was initially subjected to steepest descent energy minimization and subsequently equilibrated for ∼90 ns before starting 1-µs production simulation using Gromacs with 2-fs time steps (Pronk et al., 2013). The LINCS algorithm (Hess et al., 1997) was applied for constraining bond lengths. Electrostatic interactions were calculated with the Particle-Mesh Ewald algorithm at every step (Essmann et al., 1995). A 1.2-nm cutoff was used both for electrostatics and van der Waals interactions, with neighbors list updated every 20 steps. The simulations were performed at constant pressure of 1.0 bar with Parrinello–Rahman pressure coupling (Parrinello and Rahman, 1981) and the semi-isotropic pressure scaling, time constant of 5.0 ps, and a system compressibility of 4.5e-5 bar-1. The temperature of the system was maintained at 300°K using the extended Nosé–Hoover thermostat (Nosé, 1984). The 1-µs fully equilibrated structure was subsequently used to seed coarse-grained (CG) simulations in multicomponent lipid membranes containing either the ω-6 PUFA LIN or the saturated FA STE. STE has an 18-carbon-long acyl tail, just like LIN. The key difference, however, is that the STE tail is fully saturated (compare structures in Fig. 2). In the CG model, STE differs from LIN in the flexibility between beads 2 and 3, counting from the tail end (Fig. 2). In contrast to LIN, STE, like other saturated FAs (Liin et al., 2015), neither shifts *V*_50_ nor increases *G*_max_ of KCNQ1 (Fig. 1, C and D). Therefore, STE was used in the simulations as a negative control to LIN.

### CG MD simulations

**Figure S1.**
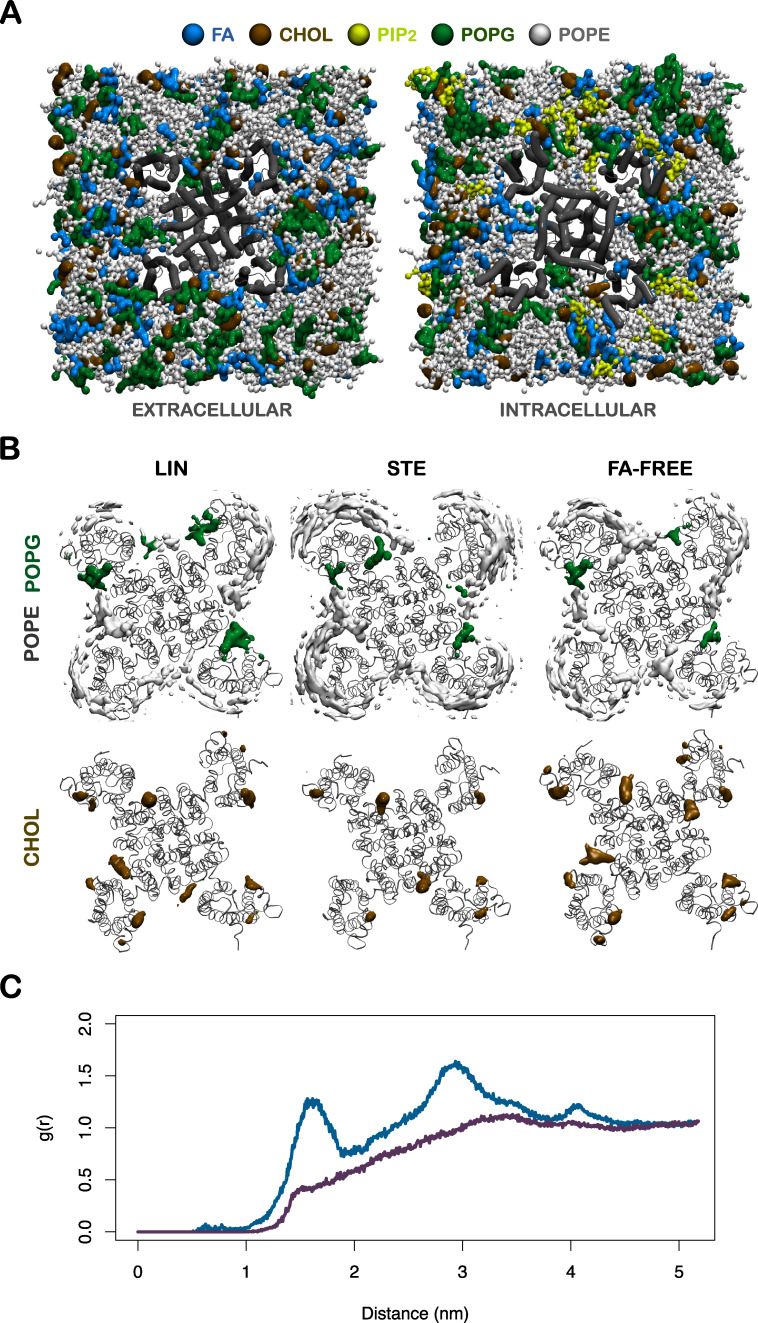
**Lipid distribution around KCNQ1 in different systems.** **(A)** An overview of the CG system offering both extracellular (left) and intracellular (right) views of the KCNQ1 channel embedded in the multicomponent membrane bilayer. The protein is displayed by the gray tube representation, and each lipid component is colored as detailed. **(B)** Contour maps of POPE (gray) and POPG (green), with threshold values at 26% (top row) and CHOL with a threshold value at 20% (bottom row) for the systems containing LIN or STE and the FA-free (FA-FREE) system. PIP_2_ is not present in the outer leaflet and was therefore not included in this analysis. **(C)** Radial distribution function of LIN (blue) and STE (purple) head groups around the KCNQ1 channel during the last 45 µs of the simulation. The radial distribution function g(r) allows a look into the distribution of particles by describing how the density varies as a function of distance from a reference particle. We observed LIN head groups peaking at ∼15 Å and 30 Å from the center of the channel, with each peak corresponding to regions I and II. The LIN and STE head group peaks differ by a factor of two or three.

The relaxed structure of the KCNQ1 channel was used as the starting structure for the CG simulations with and without FAs in the model bilayer. The channel was embedded into a lipid bilayer consisting of phosphatidylethanolamine:phosphatidylglycerol:cholesterol:FA (POPE:POPG:CHOL:FA) in a 3:1:1:1 ratio. KCNQ1 requires the presence of phosphatidylinositol 4,5-bisphosphate (PIP_2_) lipids for its function; therefore, PIP_2_ lipids were included in the inner leaflet in every bilayer simulation. The lipid composition for these systems was as follows: 307 POPE, 102 POPG, 88 CHOL, 16 PIP_2_, and 117 FA, of which 63 were distributed in the outer leaflet and 54 in the inner leaflet. Adding FA to the simulation systems did not alter the overall phospholipid distribution around the channel compared with the system free of FAs (FA-FREE; Fig. S1 B). Each system was simulated using standard Martini protocols with minor variations between systems to accommodate system-specific issues. The CG systems are circa 14.3 × 14.3 nm in the membrane plane (x and y), including 614 lipids and circa 10,300 water molecules. Simulations were performed using Gromacs (Pronk et al., 2013), with the Martini v2.2 force field parameters (de Jong et al., 2013) and standard simulation settings (de Jong et al., 2016). Briefly, all simulations were performed with a 10-fs time step, a temperature of 300°K set using a velocity-rescaling thermostat, and a time constant for coupling of 1 ps. A semi-isotropic pressure of 1 bar was maintained with the Parrinello–Rahman barostat, with a compressibility of 3·10^−4^ bar^−1^ and a relaxation time constant of 12 ps. Production runs of 50 µs were performed in the presence of position restraints applied to the backbone beads, with a force constant of 50 kJ mol^−1^ nm^−2^. The CG trajectories were clustered, and representative structures of the five-component lipid bilayer and an embedded channel were selected and back-mapped to the atomistic level using the CHARMM-GUI Martini to All-atom Converter (Jo et al., 2008). The clustering was limited to the FA head groups and performed with the built-in Gromacs tool gmx cluster, using the Jarvis–Patrick method (Jarvis and Patrick, 1973) with the distance cutoff set to 65 Å and the number of neighbors to three. The Jarvis–Patrick method adds a structure to a cluster when this structure and a structure in the cluster have each other as neighbors and they have a least a defined number of neighbors in common. The representative pose of every cluster, which is the structure with the smallest average RMSD from all other structures of the cluster, was inspected, and the frames in which FA head groups were within 10 Å of the identified high-contact and stable-contact residues at interaction sites I and II were chosen as representative structures. We selected four starting structures to seed our all-atom (AA) MD simulations on the ANTON2 platform: three structures were representative structures of the channel with LIN or STE bound to the different binding pockets mapped from the analysis of CG simulations and one was a frame with LIN fully dissociated from the channel. These structures were initially equilibrated for 500 ns with Gromacs, according to the protocol described above, with the exception of using the Berendsen barostat (Berendsen and Postma, 1984) for temperature and pressure control before starting AA runs on the ANTON2.

In addition to the above systems, two other systems were set up as described above and simulated for 50 µs: a five-component, membrane-only bilayer containing LIN and a five-component, membrane-only bilayer containing STE. The lipid composition for these systems was as follows: 572 POPE, 190 POPG, 164 CHOL, 218 FA, and 32 PIP_2_. The equilibrated 50-µs structure from each of these systems was selected as starting seeds for AA MD simulations, which were run for 1 µs with Gromacs.

### AA MD simulations

**Figure S2.**
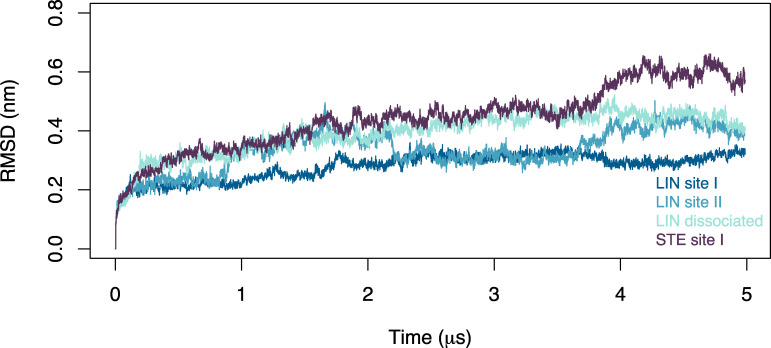
**Channel stability in AA simulations.** Backbone RMSD of the KCNQ1 channel relative to the initial structure for the four different AA simulations of the LIN and STE systems. Δ*V*_50,max_ denotes maximal effect in Δ*V*_50_.

AA production runs were performed for the following KCNQ1 systems: LIN dissociated, LIN site I, LIN site II, and STE site I. The ANTON2 software version 1.27.0 from D. E. Shaw Research was used for production runs on the purpose-built ANTON2 supercomputer (Shaw, D. E., et al. 2014. *Proceedings of the International Conference for High Performance Computing, Networking, Storage and Analysis. *https://doi.org/10.1109/SC.2014.9). The production runs were performed for 5 to 15 µs each using the CHARMM36M force field to assess structural dynamics of KCNQ1 in various membranes. The latest CHARMM36 lipid parameters were used to describe multicomponent membranes (Klauda et al., 2012). CHARMM-NBFIX LJ parameters for K^+^ and Cl^−^ (Noskov and Roux, 2008; Luo and Roux, 2010) were used to simulate counter-ion dynamics with standard TIP3P water model (Jorgensen et al., 1983). The production runs were executed in a semi-isotropic (NPaT) ensemble at temperature 315°K maintained by the Nosé–Hoover thermostat (Martyna et al., 1994). Nonbonded and long-range electrostatic interactions were evaluated every 2 and 6 fs, respectively, using the RESPA multiple–time-step algorithm (Tuckerman et al., 1992). Long-range electrostatics was calculated using the k-Gaussian Ewald method implemented to enhance performance on ANTON2 platform (Shaw, D. E., et al. 2014. *Proceedings of the International Conference for High Performance Computing, Networking, Storage and Analysis*) with a 64 × 64 × 64 grid. SHAKE was used to constrain all bonds involving hydrogen atoms. The multi-integrator (multigrator) algorithm (Lippert et al., 2013) developed in-house by D. E. Shaw Research was used for temperature and semi-isotropic pressure coupling (Shaw, D. E., et al. 2014. *Proceedings of the International Conference for High Performance Computing, Networking, Storage and Analysis*). The time step for production runs was set to 2 fs, and trajectories were saved every 500 ps. To assess the structural integrity of the KCNQ1 channel, the RMSD of the protein backbone was calculated against the initial structure for all four systems. Overall, the channels maintain stable structures, settling around an RMSD of 2.0–4.0 Å throughout the simulation time (Fig. S2).

### AA MD simulations of the R218Q/R221Q (“pseudo S4 down”) system

To construct a pseudo S4 down state of S4, we neutralized in silico the two top charges of S4 by introducing the R218Q/R221Q mutations in the model. Representative poses of LIN and STE constant binders at site I were first superimposed across each channel subunit, after which Pymol (Schrodinger, 2010) was used to introduce the R218Q/R221Q double mutation in every channel subunit. CHARMM-GUI Bilayer Maker (Jo et al., 2008) was subsequently used to prepare the system containing the protein embedded in a multicomponent lipid bilayer, according to the protocol described above. The systems, referred to as R218Q/R221Q systems, were initially equilibrated for 20 ns with position restraint acting on the bound LIN and STE molecules to allow them to relax into their placed positions, using a force constant of 1,000 kJ mol^−1^ nm^−2^. Production runs of 500 ns were performed for three replicated systems for each FA using Gromacs (Pronk et al., 2013). As control, we set up identical systems for the WT channel, referred to as “S4 up” systems, of which three replicated runs were simulated for 500 ns each. We pushed the simulations to 5 µs for two of the R218Q/R221Q systems, one for the LIN system and one for STE, in order to look at the occupancy of these FAs across different layers around binding site I. Occupancies across each 0.5-Å layer along the bilayer normal were determined by counting the total number of contacts between each FA head group and any atom on residues R218, R221, F222, Y268, F269, and L272 constituting site I.

### Analysis of FA enrichment regions and interaction sites in CG simulations

The cutoff distance between two carbon atoms for a hydrophobic interaction is 4.0 Å, and the carbon-hydrogen bond length is at ∼1.09 Å. To capture the long-range hydrophobic interactions between LIN or STE molecules and the channel, the distance cutoff between any two atoms in our systems was set to 6 Å in CG simulations, which would in effect capture any long-range molecular interactions. The cutoff distance of 6 Å is used widely in contact analysis, as it captures all important interactions between two particles and is dependent on the distance of the first minimum of the Lennard–Jones potential of the Martini force field (Marrink et al., 2007; de Jong et al., 2012; Barbera et al., 2018). The number of LIN or STE molecules within 6 Å of the channel was calculated by counting the number of negatively charged head group beads within the cutoff distance to the protein surface during the entire 50 µs of the simulation, as previous experiments have demonstrated that it is the negatively charged PUFA head group that exerts an electrostatic effect on the KCNQ1 channel (Liin et al., 2015; Larsson et al., 2018; Liin et al., 2018). Enrichment regions for LIN, STE, or other membrane components (POPE, POPG, and CHOL) were calculated by their occupancies over the last 15 µs of each CG simulation using the VolMap plugin in VMD 1.9.1 with a grid resolution of 1 Å (Humphrey et al., 1996). The 2-D occupancy maps were based on the head group beads, and the contour maps were based on entire LIN and STE molecules.

To identify the FA interaction sites in KCNQ1, we performed lipid–protein interaction analysis. An interaction is defined as when an entire (i.e., both head group and tail) lipid molecule (LIN or STE) is located within 6 Å of the channel. PUFA interaction sites were identified by two separate measures: the total interaction time and the longest lifetime. The total interaction time calculates the total time of interaction observed between each residue and any FA (could be more than one FA at a time) over the last 45 µs of the simulation, regardless of the duration of each interaction. The longest lifetime, on the other hand, is a measure of the longest-lasting contact between each residue and any FA without any interruptions in the interaction. The total interaction time and longest lifetime were averaged across the four channel subunits to account for the fourfold symmetry of the channel. Those observations that fall above Q3 (third quartile) + 1.5 interquartile range in the total interaction time and longest lifetime measurements are referred to as high-contact and stable-contact residues, respectively. Distance measurements between the FAs and the channel were calculated with the built-in Gromacs tool gmx mindist, and the total interaction time and longest lifetime measurements were done by in-house R scripts. The radial distribution functions were calculated between the LIN and STE head group beads and the channel using the built-in Gromacs tool gmx RDF.

### Analysis of FA enrichment regions and interaction sites in AA simulations

Distances between FAs and the channel for the AA simulations were calculated with the MDTraj python library. In-house R scripts were used to calculate the total interaction time made by each FA molecule over the course of the simulations. The FA molecules that had total interaction times that were in the upper quartile of total interaction time were selected for the lipid-protein analysis and are referred to as the “UQ binders.” The measurements of total interaction time and longest lifetime of each residue in the AA simulations were carried out in a manner like the CG simulation data as stated above. The FAs that stayed bound to the channel during the entire 5-µs simulation were identified by calculating the longest lifetime for each FA and are referred to as the “constant binders.” Clustering was performed with the built-in Gromacs tool gmx cluster using the GROMOS method (Daura et al., 1999), with the distance cutoff set to 5 Å. By setting a distance cutoff, the GROMOS method takes the structure with the largest number of neighbors as the centroid of the first cluster and eliminates it from the pool of structures together with all its neighbors. This procedure is repeated for all remaining structures until all structures have been designated to a cluster. The center of each cluster, which is the structure with the smallest average RMSD from all other structures of the cluster, was taken as the representative pose. Hydrogen bonds were considered to form when a hydrogen-bond donor, that is, a hydrogen atom bound covalently to an electronegative atom, was within a cutoff distance of 3.5 Å of an electronegative hydrogen-bond acceptor. As a definition of salt bridge formation, we considered a cutoff of 4 Å between N-O atom pairs.

### *Xenopus *oocyte experiments

hKCNQ1 (also called K_V_7.1; GenBank accession no. NM_000218) was in expression plasmid pXOOM. Mutations were introduced using site-directed mutagenesis (QuikChange II XL with 10 XL Gold cells; Agilent), and constructs were sequenced at the core facility at Linköping University to ensure correct sequence. Complementary RNA was prepared using T7 mMessage mMachine transcription kit (Ambion/Invitrogen). The RNA concentration was quantified using spectrophotometry (NanoDrop 2000c; Thermo Fisher Scientific). *Xenopus* oocytes were surgically isolated at Linköping University. Animal experiments were approved by the local ethics committee. Isolated *Xenopus* oocytes were injected with 50 nl of KCNQ1 RNA (50 ng RNA), and oocytes were incubated at 16°C for 2–5 d before performing two-electrode voltage clamp experiments. The two-electrode voltage clamp recordings were performed using a Dagan CA-1B Amplifier. Currents were filtered at 500 Hz and sampled at 5 kHz. The holding voltage was generally set to −80 mV. Activation curves were generally generated in steps between −80 and +60 mV in increments of 10 mV (3-s duration). The tail voltage was set to −30 mV. The control solution contained 88 mM NaCl, 1 mM KCl, 15 mM HEPES, 0.4 mM CaCl_2_, and 0.8 mM MgCl_2_. pH was set to 7.4 using NaOH. All compounds were bought from Sigma-Aldrich if not stated otherwise. Stock solutions of 100 mM LIN (9-cis, 12-cis LIN) or docosahexaenoic acid (DHA; cis-4,7,10,13,16,19–DHA), 50 mM STE, or 25 mM N-arachidonoyl taurine (N-AT; Cayman Chemical) were prepared in 99.5% ethanol. The final test solution was prepared shortly before experiments. Control or LIN or STE solution was applied using a Minipuls 3 peristaltic pump (Gilson). LIN or STE solution was applied until the effect on current amplitude reached steady state or for a minimum of 5 min. The chamber was cleaned in between each oocyte using ethanol-supplemented control solution.

### Electrophysiological analysis

Electrophysiological analysis was performed in GraphPad Prism 8 (GraphPad Software Inc.). To quantify the voltage dependence for channel opening, tail currents were measured shortly after stepping to the tail voltage and plotted against the preceding activation voltage. A Boltzmann function was fitted to the data to generate the conductance versus voltage (*G*(*V*)) curve:

$$G\left(V\right)=G_{min}+(G_{max}-G_{min})/\left\{1+\exp\left[\frac{\left(V_{50}-V\right)}{s}\right]\right\},$$ (1)

where *G_min_* is the minimal conductance, *G_max_* the maximal conductance, *V*_50_ the midpoint (i.e., the voltage at which the conductance is half the maximal conductance determined from the fit), and *s* the slope of the curve. The difference in *V*_50_ induced by LIN in each oocyte (i.e., Δ*V*_50_) was calculated to quantify the shift in the voltage dependence for channel opening. The difference in *G_max_* induced by LIN in each oocyte (i.e., Δ*G*_max_) was calculated to quantify the change in the maximal conductance. Biophysical properties of tested constructs are summarized in Table S1.

To plot the concentration dependence of the LIN-induced effect on *V*_50_ or *G_max_* as a function of the LIN concentration, the following concentration-response curve was fitted to the data:

$$\Delta Effect=\Delta Effect_{max}/{\left[1+\left(\frac{{\left[LIN\right]}_{50}}{\left[LIN\right]}\right)\right]}^{H},$$ (2)

where Δ*Effect_max_* is the maximal shift in *V*_50_ or change in *G_max_*, [LIN]_50_ the LIN concentration needed to cause 50% of the maximal effect, and H the Hill coefficient. Limited solubility of LIN at concentrations above 70 µM prevented us from quantifying the LIN effect at higher concentrations. Therefore, the Hill coefficient of the concentration-response curves was constrained to either 1 or −1 to make the fits more robust. Average values are expressed as mean ± SEM. Statistics was calculated using either one-way ANOVA followed by Dunnett’s multiple comparisons test (to compare with WT) or one-sample *t* test (to compare with a hypothetical value). P < 0.05 was considered statistically significant.

### Online supplemental material

**Figure S3.**
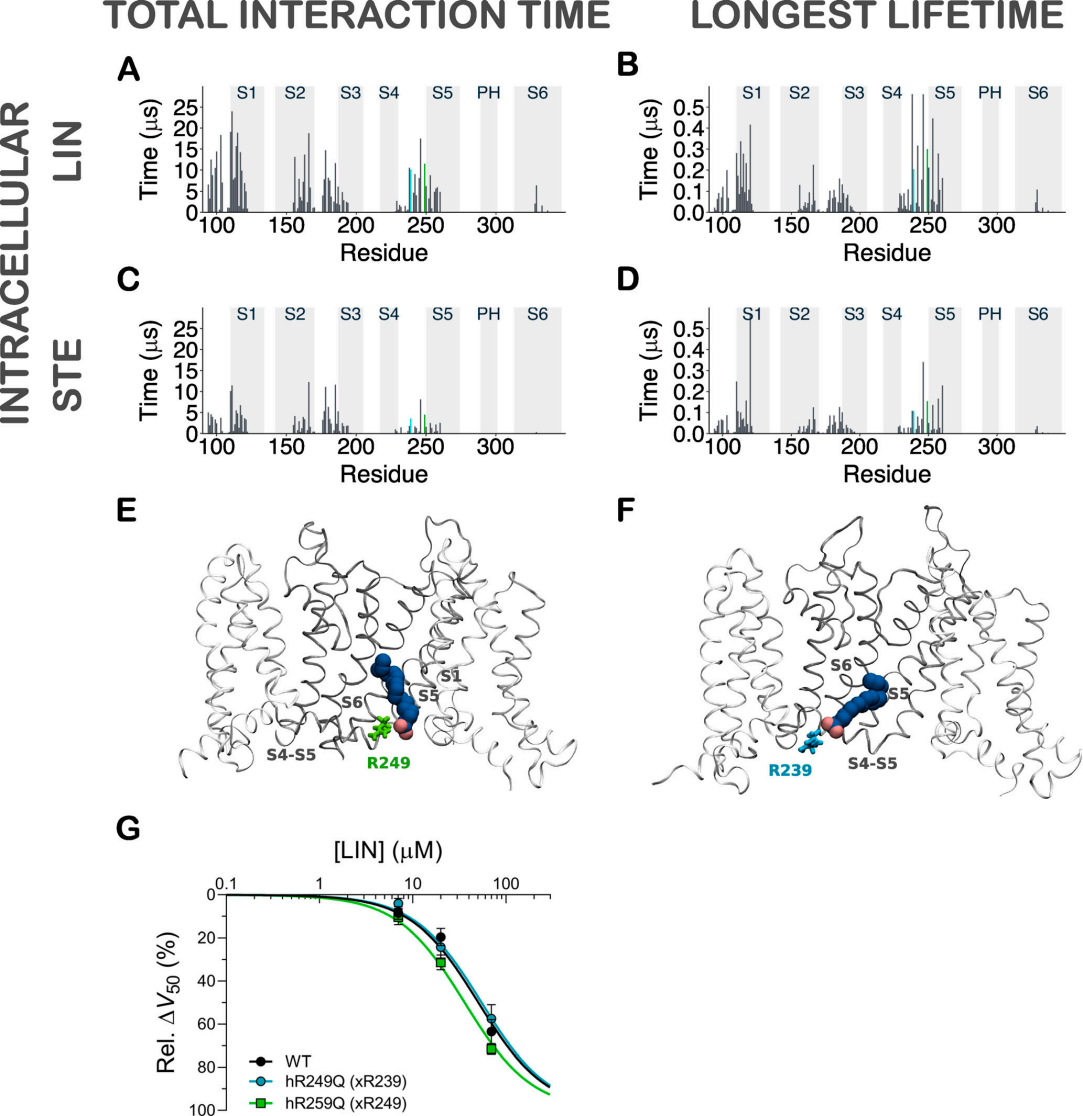
**Contact analysis for inner leaflet LIN and STE in CG simulations.** **(A)** Histogram of total interaction time of each residue in KCNQ1 for intracellular LIN. The gray highlighted boxes in the background indicate the position of the transmembrane helices. The residues identified as interacting with the LIN head group (i.e., R239 and R249) are colored according to E and F onto the histograms. **(B)** Same as in A but for longest lifetime of each residue. **(C and D)** Same as in A and B but for STE. **(E and F)** Side view of a LIN binder interacting with residues R249 and R239 located on opposite ends of the S4–S5 linker. **(G)** Experimental mutation of predicted binder residues does not impair the effect of LIN. Data shown as mean ± SEM; *n* = 5 per data point. Concentration-response curves were fitted using Eq. 2 with the Hill coefficient constrained to −1 (see Materials and methods for details). Δ*V*_50,max_ was constrained to shared values between WT and mutant to make the fits more robust, and data are shown as normalized effect (100% was defined as the maximal effect from the fit). For 70 µM LIN, Δ*V*_50_ and Δ*G*_max_ were within ±1.2 mV and ±9%, respectively (P > 0.05 with one-way ANOVA followed by Dunnett’s multiple comparisons test to compare with WT). Rel., relative.

**Figure S4.**
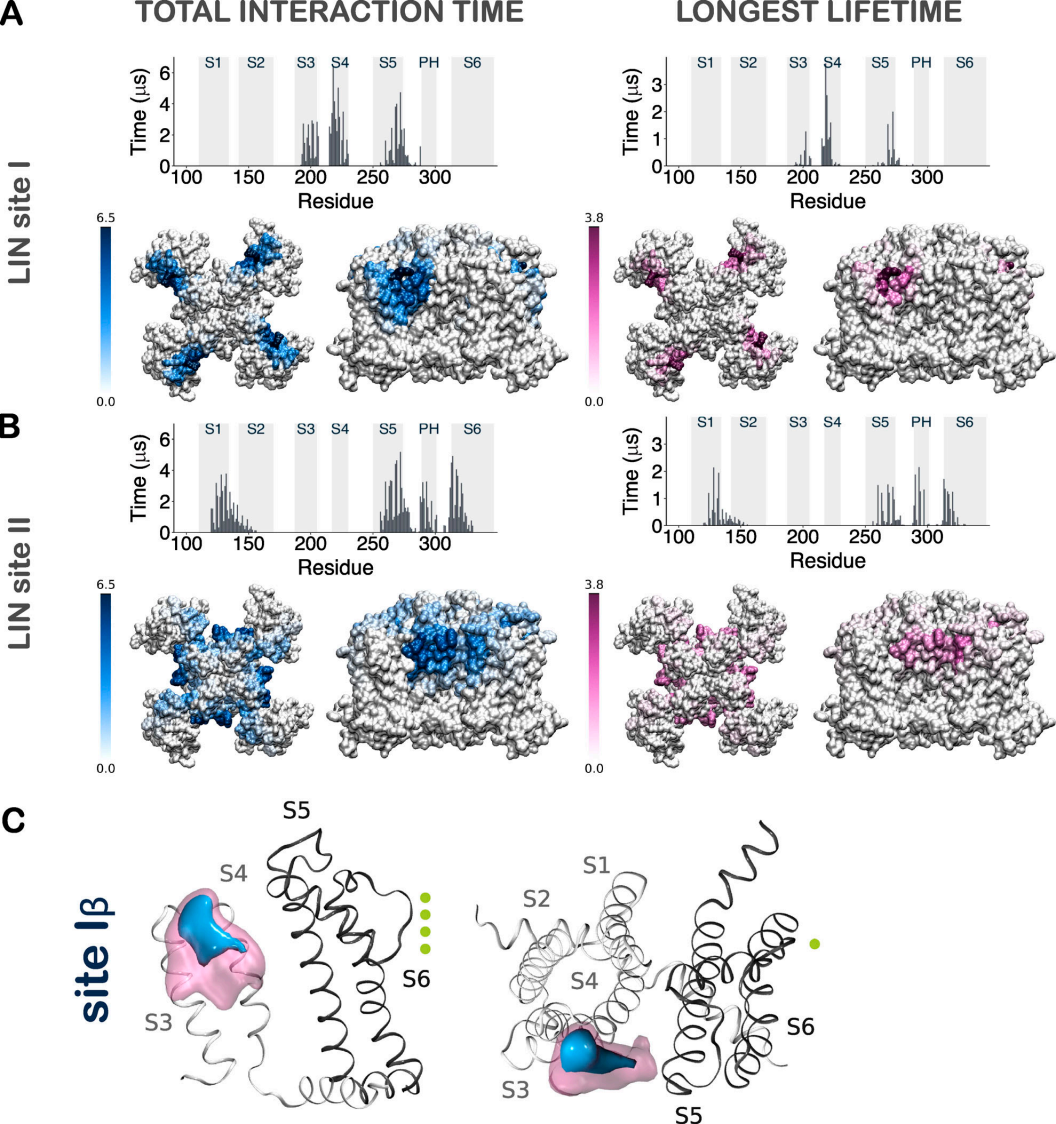
**Contact analysis for AA LIN simulations.** The LIN molecules that had a total interaction time in the upper quartile (UQ) were selected for the analysis and referred to in the text as the “LIN UQ binders.” The focus on LIN UQ binders enables direct elucidation of the long-term interactions and exclusion of random and short-lived interactions. There were in total 17 LIN UQ binders in each system. The total interaction time and longest lifetime measurements reported are averaged across the four channel subunits. We also tracked the “constant binder” LIN molecules for each specific site during the entire 5-µs simulation. There were in total two and three LIN constant binders in the LIN site I and LIN site II systems, respectively. **(A)** Left top: Histogram of total interaction time of each residue in KCNQ1 for LIN UQ binders in the LIN site I system. The gray highlighted boxes in the background indicate the position of the transmembrane helices. Left bottom: Heat maps of total interaction times in the 3-D structure of the channel shown both from top and side views. Right: Same as in A and B but for longest lifetime of each residue. The unit for the color scale is in microseconds. The longest interaction time and the longest lifetime for the LIN site I system reinforces the previously identified site I. High-contact and stable-contact residues were situated primarily on S4 and S5, but also on S3. **(B)** Same as A but for LIN UQ binders in the LIN site II system. The LIN site II simulations reveal the same interaction site II as before. The high-contact and stable-contact residues were located in S5, the pore helix and S6 of the pore domain, and S1 of the voltage-sensing domain. **(C)** 3-D contour map of the occupancy of a LIN constant binder at interaction site Iβ for AA simulations shown from side view (left) and top view (right). LIN is also observed on the S3 portion of site I, with density of a LIN constant binder concentrated between S3 and S4. This mode of binding will be referred to as site Iβ. Similar to site Iα, the density of the LIN head group is more well defined at the top of S4, as opposed to the tail density that is scattered between S3 and S4. Threshold values are set at 10% (blue) and 30% (pink) of the total time. Potassium ions in the permeation pathway are indicated as green spheres.

**Figure S5.**
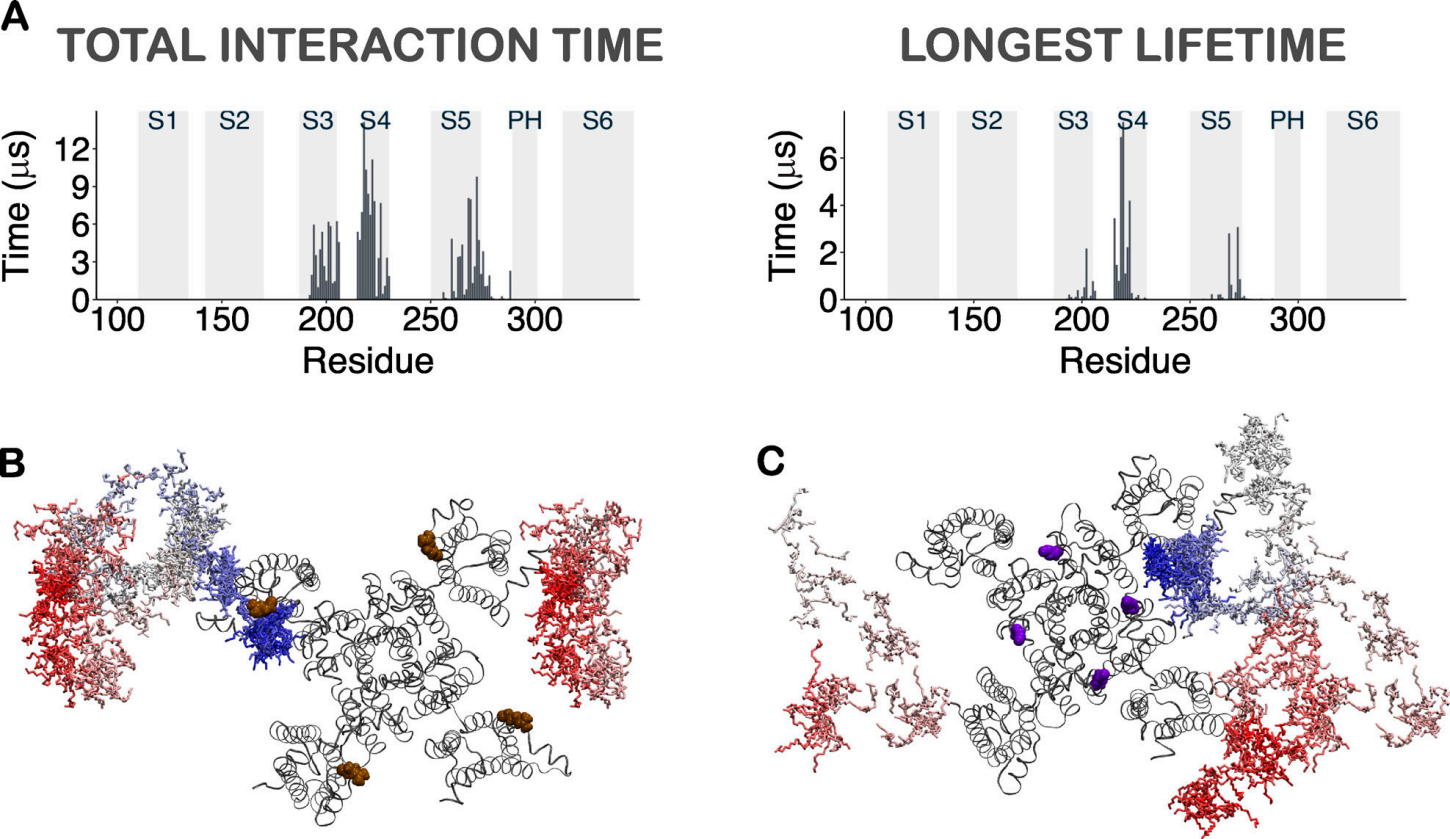
**Pattern of LIN interaction with KCNQ1 in AA simulations is preserved in longer and initial seed unbiased simulations.** **(A)** To assess whether the AA simulations we performed have allowed sufficient sampling of binding events, we continued the LIN site I system for an additional 10 µs for better sampling of the interaction sites. Contact analysis for the 15-µs AA LIN site I simulation. Histogram of total interaction time (left) and longest lifetime (right) of each residue in the transmembrane segment of KCNQ1 for LIN. The gray highlighted boxes in the background indicate the position of the transmembrane helices. The contact analysis performed for the entire 15-µs AA simulation follows the exact same trend as the initial 5 µs with the same identified constant binders keeping their close interaction with the channel. **(B and C)** To evaluate and rule out any potential convergence/initial seed system bias in AA MD simulations, a system in which no LIN molecules were in close proximity to sites I and II in the CG simulations, called LIN-dissociated, was selected as a seed system for one production run. In this control AA MD simulation, LIN molecules were allowed to diffuse in the multicomponent membrane for their preferred interaction sites. In 5-µs AA MD simulation started from LIN-dissociated state, LIN stably binds to each of the identified sites I and II. The path of a LIN molecule finding its way to site I (B) and site II (C) in the dissociated system, with red designating the starting point and blue marking the end point during a 5-µs simulation. Residues R218 and K316 are highlighted in brown and purple, respectively.

**Figure S6.**
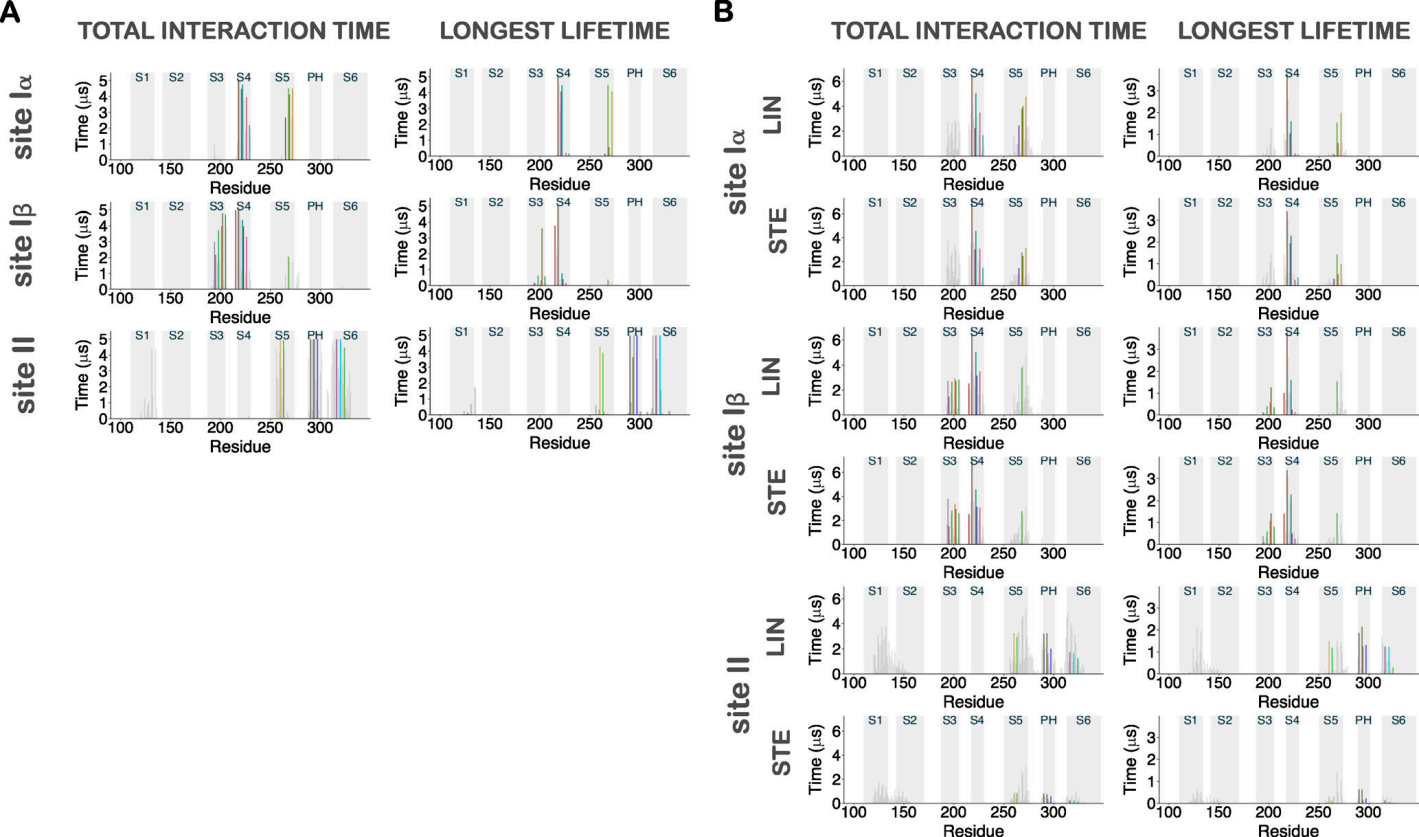
**Contact analysis for LIN constant binders in AA simulations.** **(A)** Histograms of total interaction time and longest lifetime of each residue within 6 Å of the LIN constant binders at site Iα, site Iβ, and site II. The identified binder residues (i.e., those residues that are in direct interaction with the LIN constant binder) are colored according to Fig. 4 onto the histograms. **(B)** Comparison of AA contact analysis between LIN and STE. Histograms of total interactions time and longest lifetime of each residue within 6 Å of the LIN and STE UQ binders at the identified interaction sites Iα, Iβ, and II. The identified binder residues are colored according to Fig. 4 onto the histograms. We distinctly see how the STE UQ binders fail to interact with the channel at site II, where no residues with high interaction time and lifetime were found. We do, however, observe STE to bind to both sites Iα and Iβ, albeit with notable differences in longest lifetime and total interaction time with residues from S5 compared with LIN.

**Figure S7.**
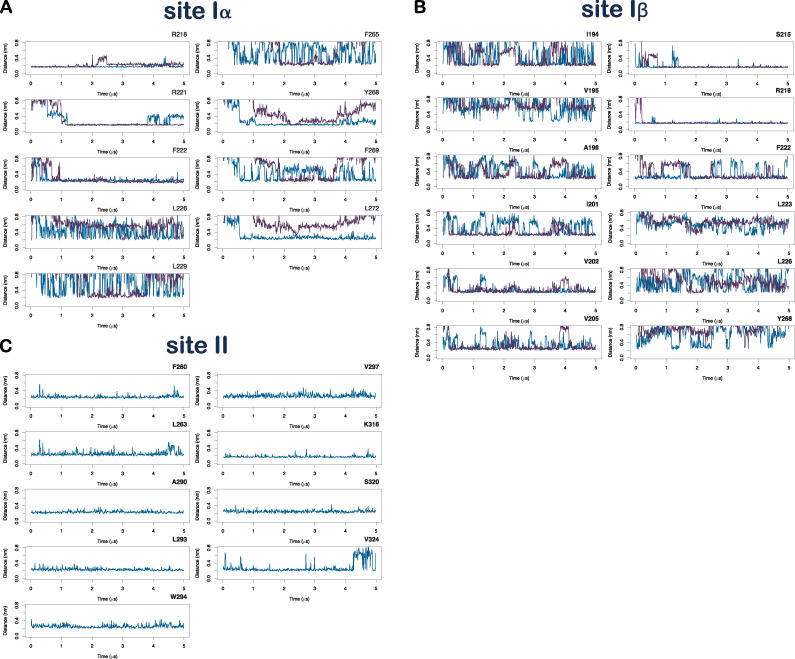
**Binder residues at sites Iα, Iβ, and II.** **(A)** Distances between the binder residues and the LIN (blue) and STE (purple) constant binders at site Iα are displayed across the 5-µs simulation period. **(B and C) **Same as in A but for site Iβ and site II, respectively. Variations in fluctuations across the distance measurements of the residues interacting with the STE and LIN constant binders are especially evident for the hydrophobic residues stabilizing the FA tails. In particular, the STE constant binders are unable to maintain a close interaction with the S5 residues Y268 and L272 in the site Iα (A). Overall, the lack of tail flexibility in STE and the stiffness of its tail prevents this saturated FA from interacting as efficiently as the more mobile unsaturated tail of LIN with the network of hydrophobic residues at site I or to fit into the more restricted cavity at site II.

**Figure S8.**
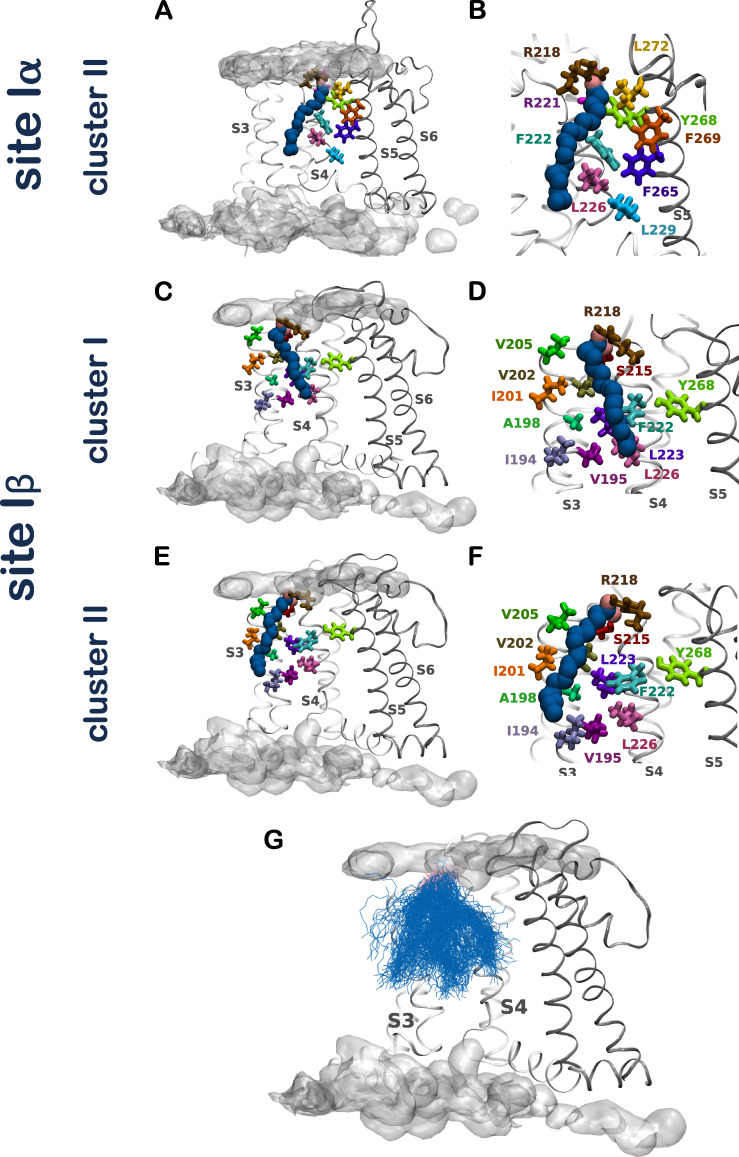
**Representative pose of a LIN constant binder at sites Iα and Iβ.** The second largest cluster at site Iα and the largest clusters at site Iβ identify overall comparable interactions between the LIN head group and R218 on S4 and a tail that sits either along S4 or more toward S3. Altogether, at either site Iα or Iβ, we observe how the LIN constant binder interacts with its head group at the top of S4, and its rather mobile tail swinging between S3, S4, and S5 results in a wide range of conformations. **(A and B)** Side view of a LIN constant binder interacting with the voltage-sensing domain at site Iα. This LIN pose is the representative pose from the second-largest cluster, representing 15% of all conformations. The LIN head group is in the same position as in the largest cluster, but with a tail that sits further along S4. **(C and D)** Side view of a LIN constant binder interacting with the voltage-sensing domain at site Iβ. This LIN pose is the representative pose from the largest cluster, containing 67% of all conformations. The binder residues at site Iβ include the basic residue R218 and the polar residue S215 on S4, which interact via salt-bridge formation and hydrogen bond with the LIN head group. The other binder residues all interact via hydrophobic interactions with the LIN tail and include I194, V195, A198, I201, V202, and V205 situated on S3; F222, L223, and L226 on S4; and the single aromatic residue Y268 on S5, which LIN interacts intermittently with (see also Fig. S7 B). **(E and F)** Same as C and D but with a LIN pose of the second-largest cluster, containing 24% of all conformations. **(G)** Overlay of all LIN binder poses at site Iβ. The LIN head group is shown in pink and the LIN tail in blue. The occupancy of the membrane lipid head groups is shown for visual purposes to denote the position of the membrane bilayer. The representative poses of LIN at this site show a head group that interacts continuously with R218 and a tail that sits either along S4 or more toward S3. Δ*V*_50,max_ denotes maximal effect in Δ*V*_50_.

**Figure S9.**
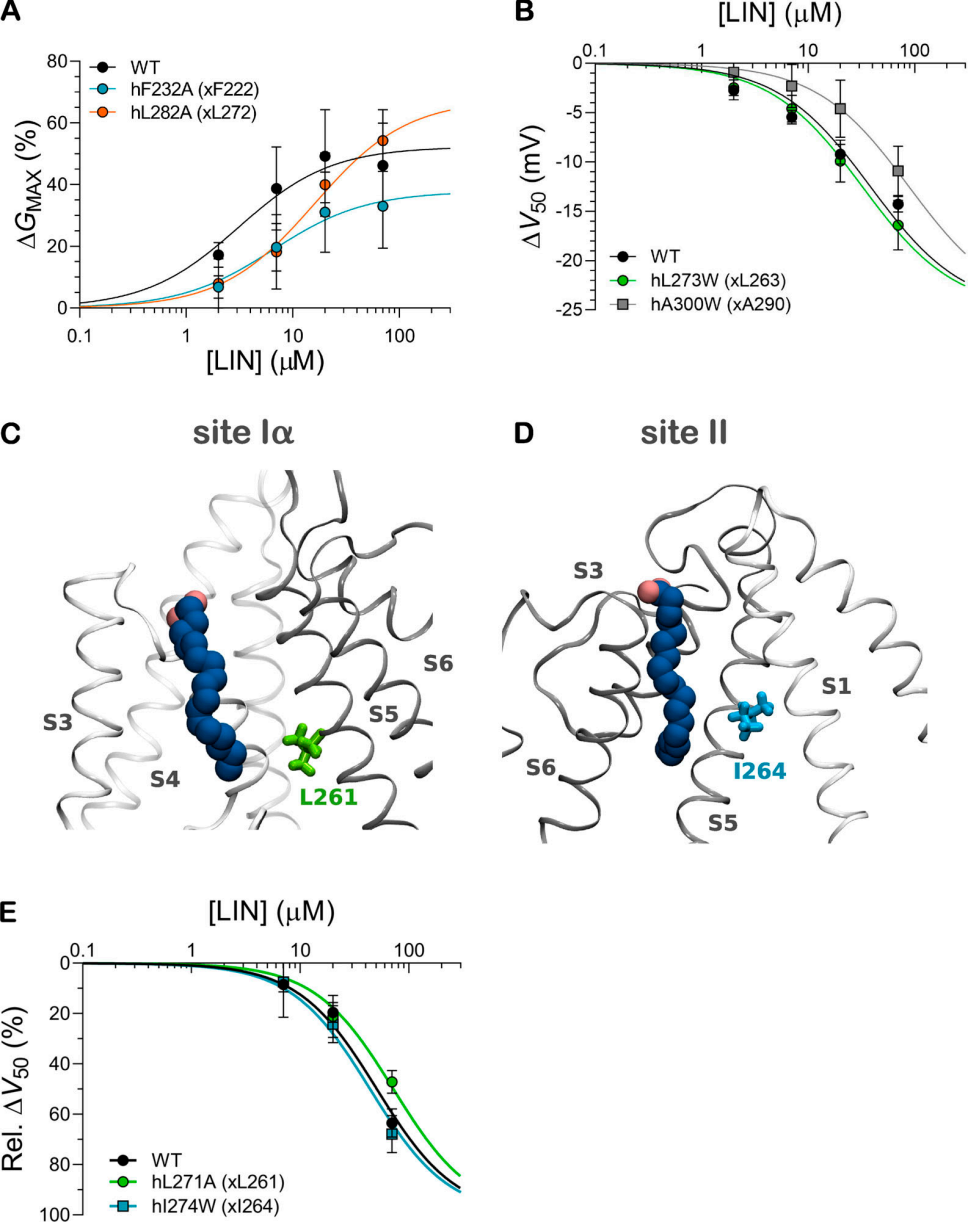
**Mutation of nearby residues at LIN interaction sites displays lack of effect.** **(A)** Concentration dependence of the LIN effect on *G*_max_ of indicated hKCNQ1 site I mutants. Data shown as mean ± SEM; *n* = 4–8 per data point. Concentration-response curves were fitted using Eq. 2 with the Hill coefficient constrained to 1 (see Materials and methods for details). Neither of the site I mutations for which *G*_max_ could be determined significantly altered the *G*_max_ effect induced by 70 µM of LIN compared with WT (P > 0.05 with one-way ANOVA followed by Dunnett’s multiple comparisons test). *G*_max_ for h279A could not be reliably determined due to unstable maximal conductance over time under control conditions. **(B)** Concentration dependence of the LIN effect on *V*_50_ of indicated hKCNQ1 site II mutants. Data shown as mean ± SEM; *n* = 6–8 per data point. Concentration-response curves were fitted using Eq. 2 with the Hill coefficient constrained to −1 (see Materials and methods for details). The hL273W mutation did not affect the ability of LIN to shift *V*_50_ (P > 0.05 with one-way ANOVA followed by Dunnett’s multiple comparisons test for 70 µM of LIN). The hA300W appeared to impair the ability of LIN to shift *V*_50_, which was detected as an apparent lower LIN affinity for the *V*_50_ effect; however, at 70 µM of LIN there was no difference compared with WT (P > 0.05 with one-way ANOVA followed by Dunnett’s multiple comparisons test). In our hands, none of the hL303F/W (xL293F/W), hL307W (xL297W), or hS330F/W (xS320F/W) mutants generated detectable K^+^ currents, and the hL307F (xL297F) mutant did not generate consistent K^+^ currents. **(C)** Side view of a LIN constant binder at site Iα and the S5 residue L261, which is near site I but predicted to not directly engage in LIN interaction. **(D)** Side view of a LIN constant binder at site II and the S5 residue I264, which is near site II but predicted to not directly engage in LIN interaction. **(E)** Experimental mutation of residues nearby to predicted binder residues does not impair the effect of LIN. Data shown as mean ± SEM; *n* = 4–5 per data point. Concentration-response curves were fitted using Eq. 2 with the Hill coefficient constrained to −1 (see Materials and methods for details). Δ*V*_50,max_ was constrained to shared values between WT and mutant to make the fits more robust, and data are shown as normalized effect (100% was defined as the maximal effect from the fit). For 70 µM LIN, Δ*V*_50_ and Δ*G*_max_ were within ±1.5 mV and ±6%, respectively (P > 0.05 with one-way ANOVA followed by Dunnett’s multiple comparisons test to compare with WT). Rel., relative.

**Figure S10.**
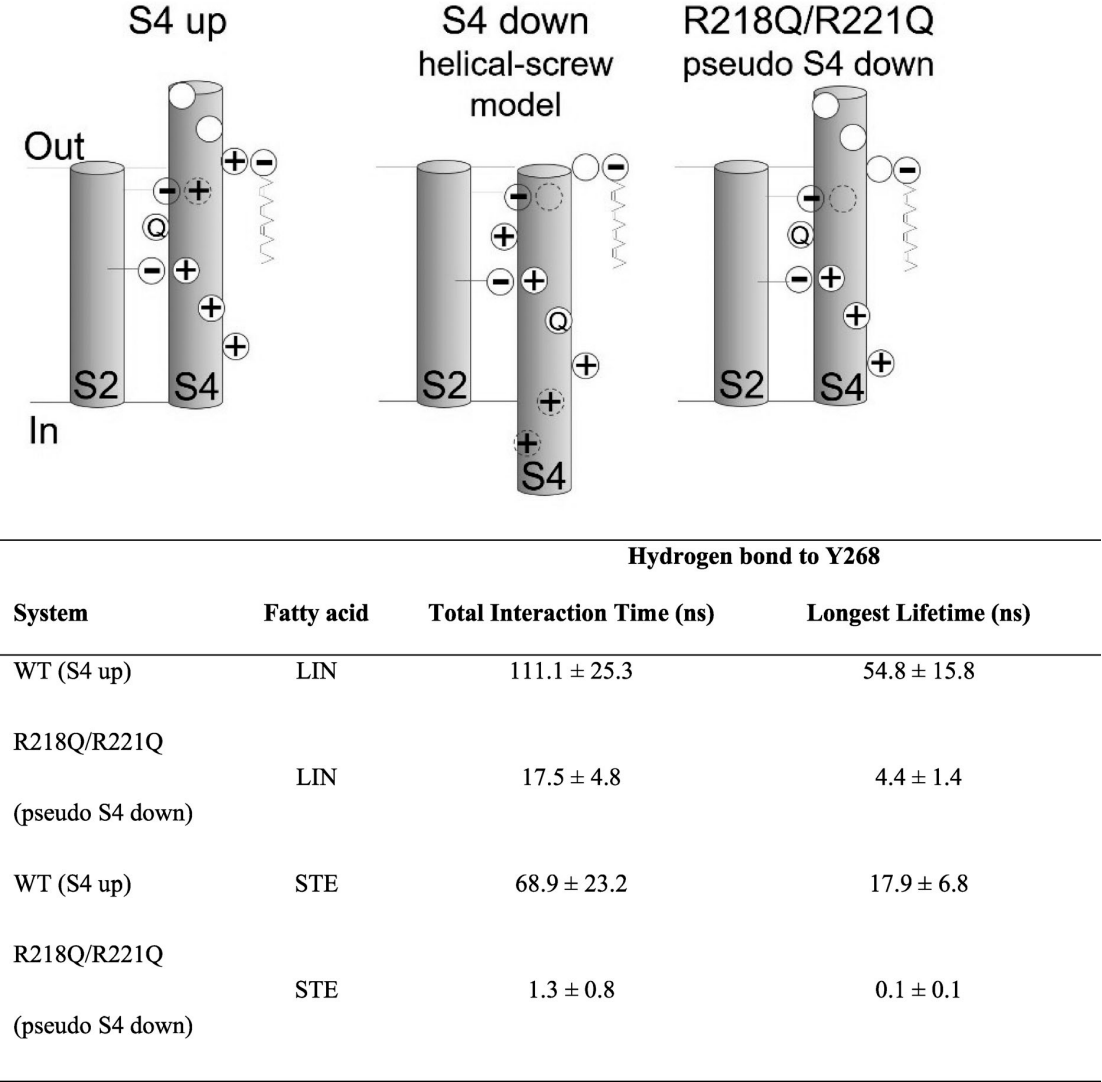
**Numerical values corresponding to** **Fig. 5, C and D.** Top: Schematic illustration comparing the S4 arginine positioning in S4 up, putative S4 down, and R218Q/R221Q (aka pseudo S4 down) states. Bottom: Total interaction time and longest lifetime of hydrogen bonds between LIN and STE head groups and the negatively charged head group of Y268 in 500 ns of AA MD of S4 up (WT) and R218Q/R221Q systems. Data shown as mean ± SEM.

**Figure S11.**
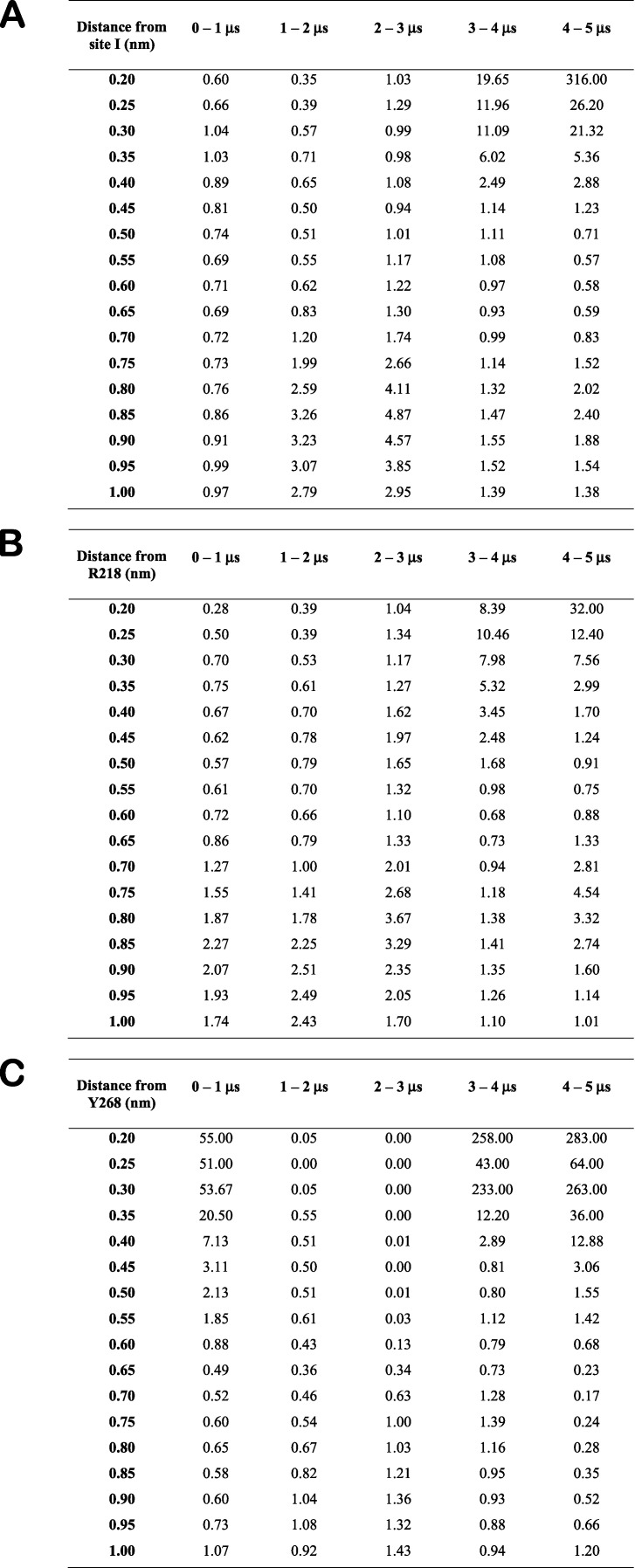
**Numerical values for differences in LIN and STE occupancy in the R218Q/R221Q system.** **(A–C)** Fold change between LIN and STE head group occupancies across layers of 0.5 Å in the horizontal plane from site I (A), R218 (B), and Y268 (C) for each consecutive 1-µs interval in 5 µs of AA MD of the R218Q/R221Q system.

**Figure S12.**
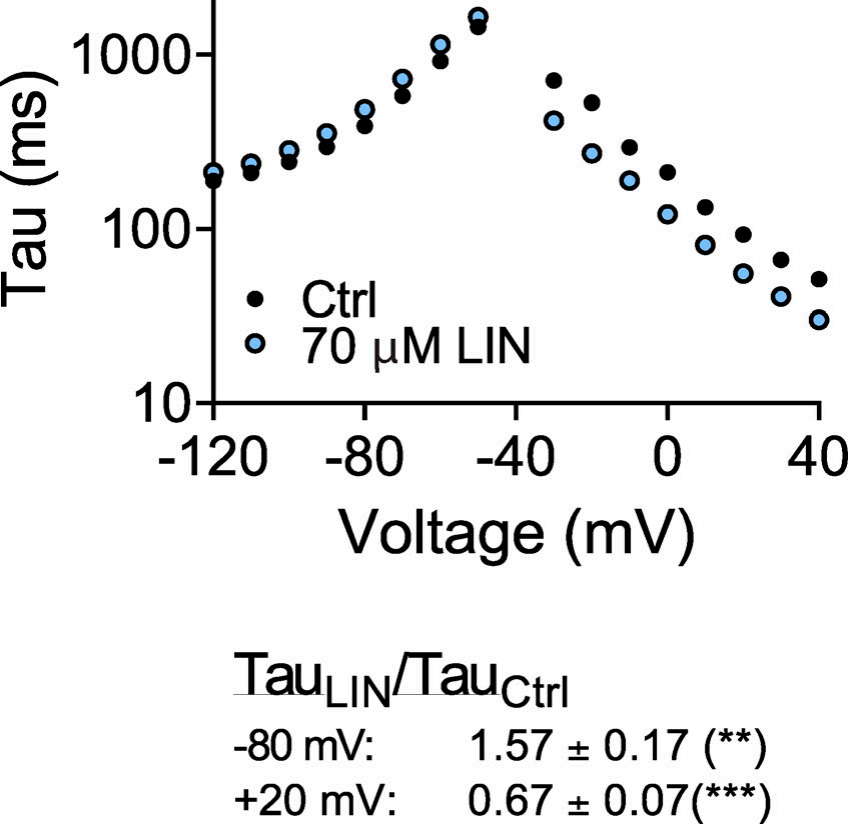
**Experimental effect of LIN on channel opening and closing kinetics.** Top: Representative effect of LIN on channel opening and closing kinetics. Experiments to determine tau for channel opening were performed by stepping from a holding voltage of −80 mV to indicated test voltages. Experiments to determine tau for channel closing were performed in high potassium solution (100 mM K^+^) by stepping from a test voltage of +40 mV to indicated test voltages. Tau was determined by fitting a single exponential function to the first second following capacitive currents. Bottom: Summary of LIN effects on closing and opening kinetics at indicated voltages. Data shown as mean ± SEM; *n* = 12. Statistics denote one-sample *t* test compared with a hypothetical value of 1. **, P < 0.01; ***, P < 0.001. Ctrl, control.

**Figure S13.**
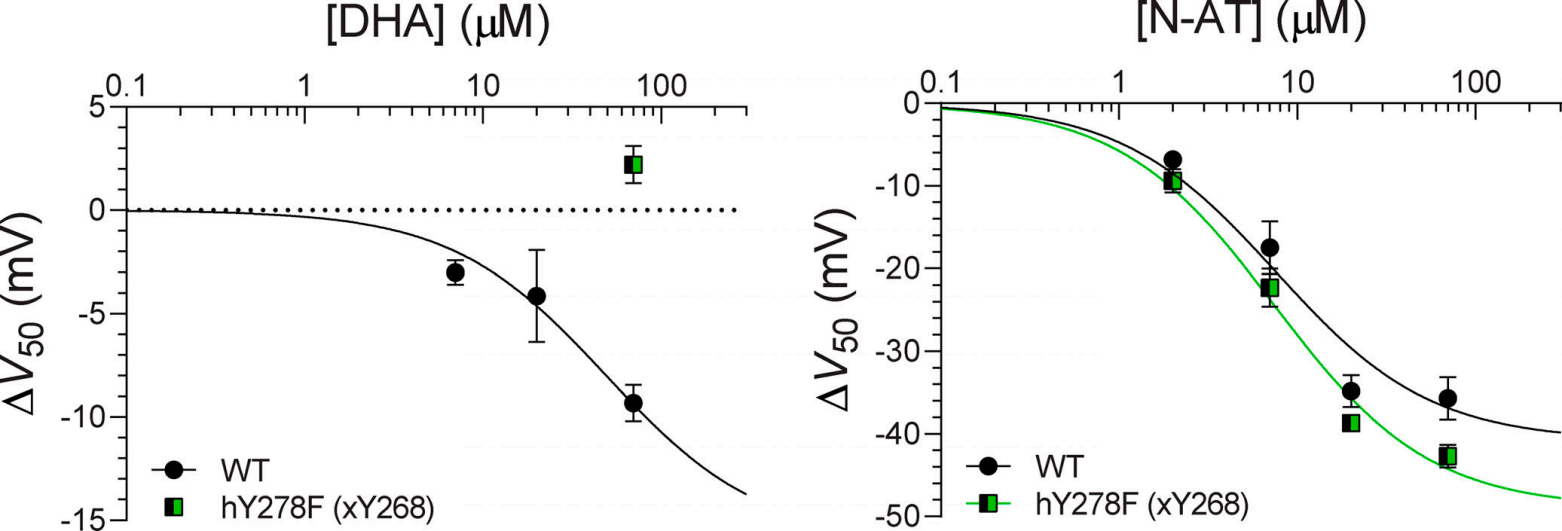
**Impact of Y268 for experimental DHA and N-AT effects.** Experimental mutation of tyrosine hY278 (xY268) impairs the ability of the PUFA DHA (left) but not the PUFA analogue N-AT (right) to shift *V*_50_ of KCNQ1. Data shown as mean ± SEM; *n* = 3–7 per data point. Concentration-response curves were fitted using Eq. 2 with the Hill coefficient constrained to −1 (see Materials and methods for details).

Fig. S1 shows the lipid distribution around KCNQ1 in different systems. Fig. S2 shows channel stability in AA simulations. Fig. S3 presents contact analysis for inner leaflet LIN and STE in CG simulations. Fig. S4 presents contact analysis for AA LIN simulations. Fig. S5 shows that the pattern of LIN interaction with KCNQ1 in AA simulations is preserved in longer and initial seed unbiased simulations. Fig. S6 presents contact analysis for LIN constant binders in AA simulations. Fig. S7 shows binder residues at sites Iα, Iβ, and II. Fig. S8 illustrates the representative pose of a LIN constant binder at sites Iα and Iβ. Fig. S9 shows that the mutation of nearby residues at LIN interaction sites displays lack of effect. Fig. S10 presents numerical values corresponding to Fig. 5, C and D. Fig. S11 presents numerical values for differences in LIN and STE occupancy in the R218Q/R221Q system. Fig. S12 shows the experimental effect of LIN on channel opening and closing kinetics. Fig. S13 shows the impact of Y268 for experimental DHA and N-AT effects. Video 1 shows the top view of the 5-µs AA simulation of LIN in the site I system. Video 2 shows the top view of the 5-µs AA simulation of LIN in the site II system. Table S1 lists the biophysical properties of KCNQ1 mutants.

## Results

We studied the interaction of the ω6 PUFA LIN or saturated FA STE with the KCNQ1 channel by combining long-time-scale CG MD and microseconds-long AA MD simulations in a multicomponent membrane bilayer with a lipid composition of POPE, POPG, CHOL, and PIP_2_ (inner leaflet only; Fig. S2, A and B) and PUFAs added to both leaflets. The levels of the different lipids were chosen to be comparable to the concentrations in simplified models of the cardiomyocyte membrane (Marrink et al., 2019). 50-µs-long CG simulations allowed us to directly map putative PUFA–channel interaction sites by evaluating total interaction time and the longest lifetime of each residue. To further characterize these sites, we proceeded with 5–15-µs-long AA MD simulations and identified the so called constant binders, which are molecules that stay bound to the putative interaction sites during the entire simulation, and binder residues, which are residues that interact in a direct and continuous manner with the constant binder. Based on our previous experimental findings that residues in the extracellular part of the channel are important for PUFA activation of the KCNQ1 channel (Liin et al., 2015; Larsson et al., 2018; Liin et al., 2018), we focused our analysis on PUFA–channel interactions in the outer leaflet of the lipid bilayer. As a control, we further tested putative PUFA binding sites in the inner leaflet (see LIN–KCNQ1 interactions).

### LIN, but not STE, is highly enriched in two distinct regions of the KCNQ1 channel

50-µs CG MD simulations of KCNQ1 in a multicomponent lipid bilayer, with or without LIN, were performed to map the lipid distribution around the channel. The number of LIN molecules within 6 Å of the KCNQ1 channel remained nearly constant throughout the 50-µs-long simulation, with an average of 6.6 LIN molecules within 6 Å around the entire channel (Fig. 2 A). 2-D occupancy maps of the LIN head group in different layers along the z axis of the membrane (bilayer normal) revealed two distinct high-occupancy regions in the different layers across all four voltage-sensing domains (Fig. 2 B). A high-occupancy layer marked by R218 in the KCNQ1 model contributes to binding region I formed by residues from S3, S4, and S5 helices (Fig. 2 B, left). A layer marked by K316 in the KCNQ1 model defines binding region II, composed of residues from S1 and S6 helices (Fig. 2 B, right). These highly LIN-enriched binding regions are clearly visible in the contour map of occupancy of entire LIN molecules, highlighting LIN enrichment at both regions I and II across at least three voltage-sensing domains (Fig. 2 C). The two sites in proximity of R218 and K316, respectively, are consistent with previous experimental findings of two independent PUFA effects on KCNQ1 reliant on charge conservation in these positions (Liin et al., 2018). CG MD simulations identified important quantitative differences in STE enrichment at the KCNQ1 channel surface: the number of STE compared with LIN molecules near the channel differed by a factor of two to three (Fig. 2 and Fig. S1 C), with no enrichment of STE at region II and limited enrichment at region I (Fig. 2, E and F).

### CG MD simulations identify two main interaction sites for LIN on KCNQ1

**Figure 3.**
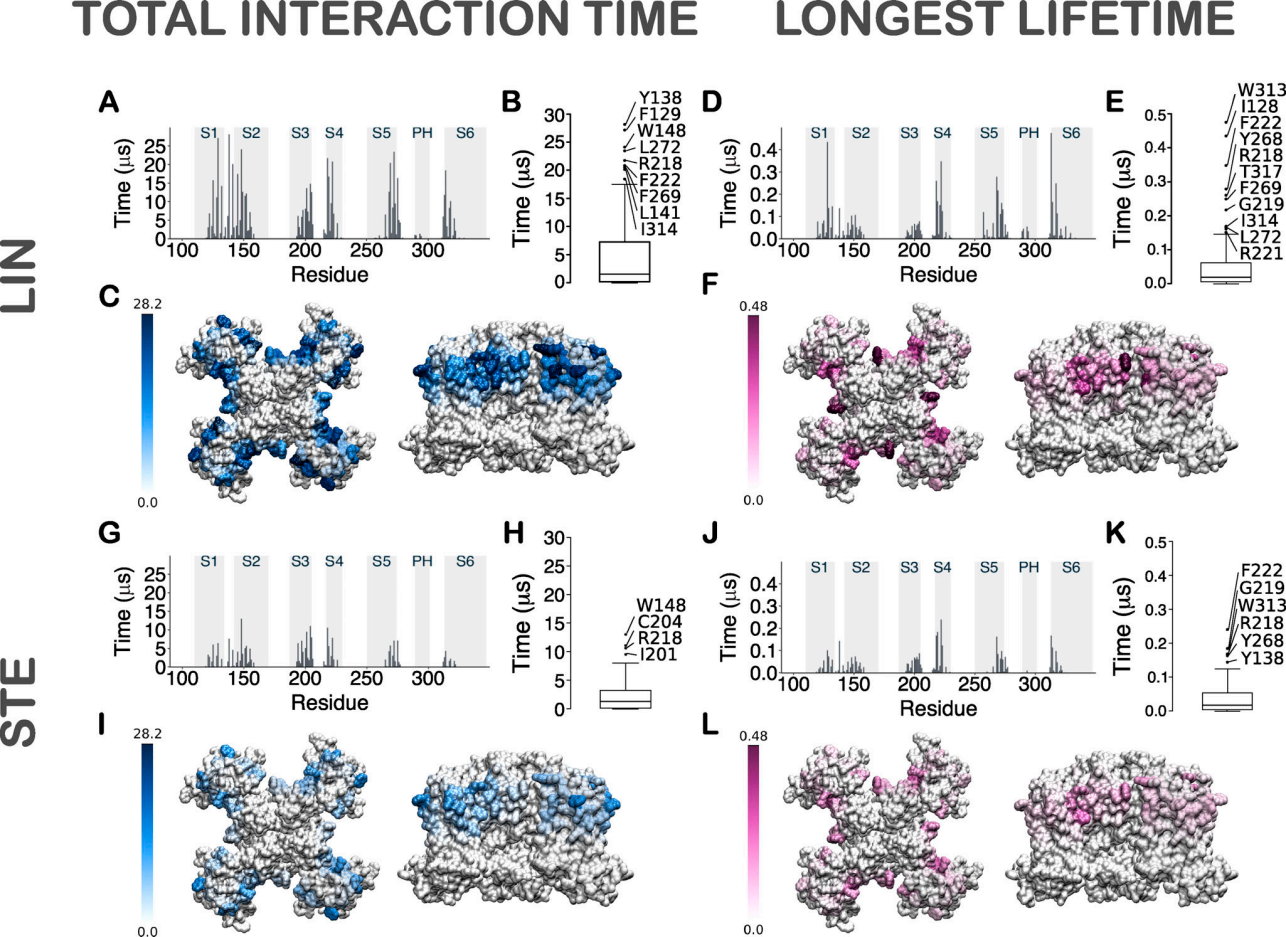
**Contact analysis for LIN and STE in CG simulations.** **(A)** Histogram of total interaction time of each residue in KCNQ1 for LIN. The gray highlighted boxes in the background indicate the position of the transmembrane helices. The total interaction times reported cover the simulation period of 5–50 µs, since convergence of the number of LIN molecules within 6 Å of the channel is achieved after 5 µs into the simulation. The total interaction time for each residue has been averaged across the four channel subunits to account for the fourfold symmetry of the channel. **(B)** Box plots showing the total interaction time for the residues, with those that fall above Q3 + 1.5 interquartile range in the total interaction time (i.e., high-contact residues) labeled. **(C)** Heat maps of total interaction times in the 3-D structure of the channel shown both from a top and a side view. The unit for the color scale is in microseconds. **(D–F)** Same as in A–C but for longest lifetime of each residue. As with the total interaction time calculations, the longest lifetime measurements are based on the last 45 µs of the simulation and are averaged across the four channel subunits. **(G–L)** Same as in A–F but for STE. The only common high-contact residue between the two FAs in the total interaction time analysis was R218 in S4 at site I (H). However, instead of the other residues that formed site I for LIN, high-contact residues for STE included W148 in S2 and I201 and C204 in S3 (H). While site I was rarely occupied in the STE simulations, STE at site II could not be identified in the total interaction time analysis (G–I). When comparing the longest lifetime residues for the two FAs, similarly to LIN, residues R218, G219, and F222 in the S3–S4 loop and Y268 in S5 form site I for STE. However, in contrast to LIN, for STE, F269 and L272 did not contribute to site I and I314 and T317 in S6 and I128 in S1 did not contribute to site II. For STE, only the upper S6 residue W313 and S1 residue Y138 form interaction site II.

To further characterize the identified regions of PUFA–channel interaction, we measured the total interaction time of each residue with every LIN molecule (interaction = a LIN within 6 Å of the residue) on the external leaflet of the membrane bilayer (Fig. 3 A), an approach that allowed us to identify two distinct sites defining LIN–channel interaction (Fig. 3, B and C). High-contact residues located on the upper parts of transmembrane segments S4 and S5 constitute interaction site I, which forms a pocket between S4 and S5. The high-contact residues at site I include R218 and F222 in S4 and F269 and L272 in S5. High-contact residues located in the upper parts of transmembrane segments S6 and S1 constitute interaction site II, which is formed by a cavity in between these two helices. The high-contact residues at site II are I314 in S6 and F129 and Y138 in S1. Another measure of PUFA–channel interaction is the residues–LIN interaction with the longest lifetime for each residue (Fig. 3 D). The longest lifetime measurements distinguished long-lived, stable contacts from short-lived, but frequent, low-affinity contacts and highlight the regions of higher affinity binding on the channel surface (Fig. 3, E and F). The stable-contact residues in site I include R218, G219, R221, and F222 in S4 and Y268, F269, and L272 in S5. At interaction site II, the stable-contact residues are W313, I314, and T317 in S6 and I128 in S1.

Thus, the combined approach of measuring total interaction time with the longest lifetime of interacting residues of KCNQ1 enabled us to identify two plausible PUFA–channel interaction sites within the regions of PUFA enrichment. These two regions are in excellent spatial agreement with regions I and II identified by the occupancy maps. In contrast, for STE the total interaction time as well as the longest lifetime were reduced by at least a factor of 2 compared with that of LIN, also with differences in which residues constitute high-contact residues (Fig. 3, G–L).

### LIN–KCNQ1 interactions in inner leaflet do not contribute to functional effects

Although our analysis was focused on interactions in the outer leaflet, the CG MD simulations were performed with either LIN or STE also in the inner leaflet. In analogy with interactions in the outer leaflet, the total interaction time as well as the longest lifetime were longer for LIN compared with STE in the inner leaflet (Fig. S3, A–D). Clustering of LIN molecules interacting with the inner part of KCNQ1 identified R239 and R249 as putative interaction residues with the LIN head (Fig. S3, E and F). However, two-electrode voltage clamp experiments on hR249Q (xR239) and hR259Q (xR249) mutants did not support any functional roles of potential LIN interactions with these residues for LIN effects: both mutants responded to LIN with induced *V*_50_ and *G*_max_ effects comparable to those of WT (Fig. S3 G and figure legend). These data suggest that although LIN shows more prominent accumulation than STE near KCNQ1 in the inner leaflet, LIN interactions with KCNQ1 residues in the inner leaflet do not contribute significantly to the functional effects on KCNQ1. This finding highlights the importance of functional testing of putative binding sites identified in simulations.

### AA simulations resolve atomistic details for the interaction of the LIN head and tail with KCNQ1 at site I

**Figure 4.**
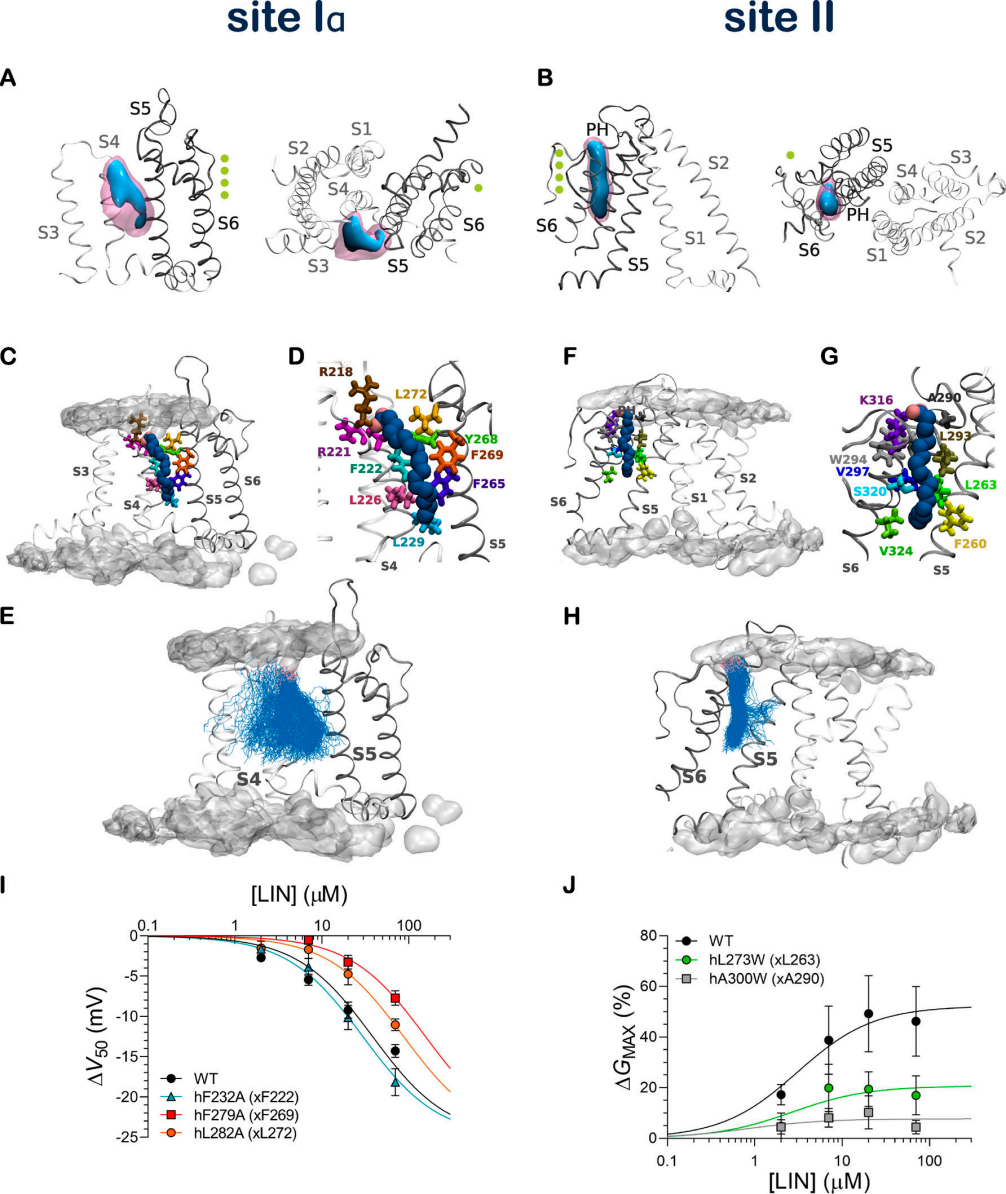
**LIN binds at interaction sites through a network of charged and hydrophobic residues.** **(A and B)** 3-D contour maps of the occupancy of a LIN constant binder at site Iα (A) and site II (B) are shown from side view (left) and top view (right). Threshold values are set at 10% (blue) and 30% (pink) of the total time. PH denotes pore-helix. Potassium ions in the permeation pathway are indicated as green spheres. **(C and D)** Side view of a LIN constant binder interacting with the voltage-sensing domain at site Iα. **(E)** Overlay of all LIN constant binder poses at site Iα. The LIN head group is shown in pink and the LIN tail in blue. **(F and G)** Side view of a LIN constant binder interacting with the pore domain at site II. **(H)** Overlay of all LIN binder poses at site II. The occupancy of the membrane lipid head groups is shown for visual purposes to denote the position of the membrane bilayer. **(I and J)** Mutation of predicted binder residues impairs the effect of LIN. **(I)** Concentration dependence of the LIN effect on *V*_50_ of indicated hKCNQ1 site I mutants. Data shown as mean ± SEM; *n* = 4–8 per data point. Concentration-response curves were fitted using Eq. 2 with the Hill coefficient constrained to −1 (see Materials and methods for details). Δ*V*_50,max_ was constrained to −25 mV to make the fits more robust. **(J)** Concentration dependence of the LIN effect on *G*_max_ of indicated hKCNQ1 site II mutants. Data shown as mean ± SEM; *n* = 6–8 per data point. Concentration-response curves were fitted using Eq. 2 with the Hill coefficient constrained to 1 (see Materials and methods for details). Δ*V*_50,max_ denotes maximal effect in Δ*V*_50_.

We next examined LIN and STE interactions with KCNQ1 at sites I and II on the atomistic level. We first focused the analysis on LIN interaction with KCNQ1 in two AA simulations of systems, where CG MD frames in which LIN molecules that were in the vicinity of each of the two identified interaction sites were converted to atomistic structures and were used to seed 5-µs AA MD simulations (referred to as LIN site I and site II systems in Materials and methods). Long-lived interactions between LIN molecules and KCNQ1 over the course of the AA MD simulations were monitored by determining the longest interaction time and longest lifetime of LIN molecules in the upper quartile. This analysis of upper-quartile LIN molecules reinforces the previously identified sites I and II. Fig. 4 A displays typical density of LIN at site I, with the LIN head group concentrated at the top of S4, whereas the density associated with the mobile tail is distributed with a higher intensity near S5 (see also Fig. S4 A and Video 1). We refer to this location of LIN molecules on the S5 side of S4 as site Iα. At site I, we also observed LIN molecules on the S3 side of S4, referred to as site Iβ, with the density of the LIN head group at the top of S4 and tail density scattered between S3 and S4 (Fig. S4 C). The LIN density at site II is concentrated in the cavity formed by S1, S5, and S6 in a similar manner as in the CG simulations for both the head and tail groups of the molecule (Fig. 4 B, Fig. S4 B, and Video 2).

**Video 1.** **Top view of the 5-µs AA simulation of LIN in the site I system.** The LIN UQ binders, that is, those LIN molecules that make up the upper quartile of total number of channel interactions, are displayed with the LIN head group shown in pink and the tail in blue. Residues R218 and K316 are colored in brown and purple, respectively. For the sake of visual clarity, the other lipids in the systems are not displayed. Playback speed, 25 frames/s.

**Video 2.** **Top view of the 5-µs AA simulation of LIN in the site II system.** The LIN UQ binders, that is, those LIN molecules that make up the upper quartile of total number of channel interactions, are displayed with the LIN head group shown in pink and the tail in blue. Residues R218 and K316 are colored in brown and purple, respectively. For the sake of visual clarity, the other lipids in the systems are not displayed. Playback speed, 25 frames/s.

To ensure sufficient sampling in the AA MD simulations, we extended the AA simulations for the LIN site I system for an additional 10 µs, which showed a similar pattern of LIN interaction with KCNQ1 (Fig. S5 A). To rule out any potential initial seed system bias in AA MD simulations, one CG frame with no LIN molecules in close proximity of site I and II was selected as a seed system for a 5-µs-long AA MD simulation. In this system, started from a LIN-dissociated state, LIN diffuses and stably binds to each of the identified sites I and II (Fig. S5, B and C).

We next turned our attention to three LIN constant binders, one at each distinct interaction site (sites Iα, Iβ, and II), to identify channel residues important for LIN interaction. Through distance measurements of each residue identified as interacting with the constant binders, we were able to discern between proximal residues (i.e., those residues that are merely within 6 Å of the binder but do not interact directly with LIN) and binder residues (i.e., residues that are in direct contact with the constant binder LIN molecule and form important interactions of either an electrostatic or hydrophobic nature; Fig. S6 and Fig. S7). The promiscuous LIN tail required clustering of all the LIN binder conformations to allow for selection of representative poses of the LIN constant binder. The representative pose of the largest cluster of LIN binder conformations at site Iα (encompassing 70% of all LIN conformations) shows a LIN constant binder that sits snugly in between S4 and S5: its negatively charged head group forms direct interaction with R218, while its curled-up tail is lined up along residues situated on S4 and S5 deep into the membrane (Fig. 4, C and D). The binder residues encompass the basic residues R218 and R221 on S4 and the aromatic residue Y268 on S5, which through a network of salt bridges and hydrogen bonds stabilize the LIN head group in between S4 and S5. Meanwhile, the mobility of the tail allows for hydrophobic interactions with residues F222, L226, and L229 on S4 and the S5 residues F265, F269, and L272 (Fig. 4, D and E; and Fig. S7 A). The representative poses from additional clusters at site Iα and Iβ display an overall comparable pattern of interaction between the LIN and KCNQ1 (see Fig. S6, Fig. S7, and Fig. S8 for further details). Based on the similarity of interactions between the LIN head group and the top arginines of S4 in sites Iα and Iβ, we consider these sites as variants of site I with anticipated similar effects on KCNQ1 voltage dependence.

### AA simulations resolve atomistic details for the interaction of the LIN head and tail with KCNQ1 at site II

At site II, the properties of the binder residues lining this pocket are, as for site I, of either charged, aromatic, or hydrophobic nature (Fig. S7 C). At site II, the LIN constant binder conformations form one large cluster encompassing 98% of all conformations. The representative pose displays a LIN constant binder that sits comfortably in the cavity formed by S5, the pore helix, and S6 (Fig. 4 F). While the head group interacts mainly with K316 on S6 via a salt bridge, the tail forms a number of hydrophobic interactions with residues F260 and L263 on S5; A290, L293, W294, and V297 on the pore helix; and S320 and V324 on S6 (Fig. 4, F and G; and Fig. S7 C). Here, we see how the LIN constant binder maintains largely the same conformation and binds quite stably in this narrow interaction site (Fig. 4 H).

Altogether, the detailed probing of LIN–KCNQ1 interactions in the AA simulations has enabled a finer characterization of the LIN interaction sites. A common feature found for all sites is the bi-component mechanism of specific interactions: the stable binding relies on LIN head group electrostatic interactions with a positively charged residue located at the top of the channel close to water–membrane interface, while the LIN tail interacts with a network of hydrophobic residues embedded deeper into the membrane. In contrast, we distinctly see in AA simulations how the STE fails to interact with the channel at site II (Fig. S6 B), and is unable to maintain close interactions with the S5 residues Y268 and L272 at site I (Fig. S7). Overall, the stiffness of the STE tail prevents this saturated FA from interacting as efficiently as the more mobile unsaturated tail of LIN with the network of hydrophobic residues at site I or from occupying the more restricted cavity at site II (Fig. S6 and Fig. S7). The involvement of aromatic residues in the stabilization of the unsaturated LIN tail is in line with the recent observation of a specific interaction between unsaturated tails and aromatic residues of a sensor protein (Ballweg et al., 2020). Using an array of biophysical and modeling techniques, Ballweg et al. (2020) showed that the presence of unsaturated alkyl tails in lipids stabilizes lipid–aromatic group interactions via, presumably, specific CH-π stacking interactions. At the same time, the presence of saturated lipid tails is a destabilizing factor for protein–lipid interactions.

### Electrophysiology experiments verify LIN tail–KCNQ1 interactions predicted from simulations

Previous electrophysiology experiments have shown that the basic residues on S4 and S6 identified as binder residues for the PUFA head in the AA simulations (i.e., R218 and R221 on S4 and K316 on S6) are important for PUFA effects on *V*_50_ and *G*_max_, respectively (Liin et al., 2018). However, the importance of the predicted binder residues for the PUFA tail from the AA simulations remains experimentally unstudied. We therefore used two-electrode voltage clamp electrophysiology on KCNQ1 to experimentally test the effect of mutating specific binder residues.

For site I, we focused on F222, F269, and L272, which interacted most consistently with the LIN tail (Fig. S7 A). We reasoned that reducing the size of the side chain of these hydrophobic residues through substitution with the hydrophobic but smaller alanine may impair the ability of the LIN tail to interact with these residues to enable the shift in *V*_50_. Whereas alanine mutation of hF232 (xF222) did not affect the ability of LIN to shift *V*_50_, alanine mutation of hF279 (xF269) or hL282 (xL272) impaired the ability of LIN to shift *V*_50_, which was detected as an apparent lower LIN affinity for the *V*_50_ effect seen as a shift in the concentration-response curve toward higher LIN concentrations (Fig. 4 I). At 70 µM of LIN, the hF279A mutant shifted *V*_50_ by only −7.7 mV compared with −14.3 mV WT (P < 0.05 with one-way ANOVA followed by Dunnett’s multiple comparisons test). In contrast, neither of the site I mutants for which *G*_max_ could be determined significantly altered the ability of LIN to increase *G*_max_ (Fig. S9 A).

For site II, we focused on L263, A290, L293, V297, and S320, which interacted consistently with the LIN tail (Fig. S7 C). Because of the narrow nature of site II, we reasoned that increasing the size of the side chain of these residues through substitution with hydrophobic but bulkier phenylalanine or tryptophan may impair the ability of the LIN tail to interact with this site, a property of PUFA essential for an increase in *G*_max_. In general, mutation of this site was poorly tolerated by the channel, but the effect of mutation of hL273 (xL263) and hA300 (xA290) could be experimentally tested. Tryptophan mutation of hL273 (xL263) and hA300 (xA290) impaired the ability of LIN to increase *G*_max_, which was detected as a smaller maximal effect induced by LIN (Fig. 4 J). At 70 µM of LIN, the hL273W mutant increased *G*_max_ by <20% compared with 49% WT (P < 0.05 with one-way ANOVA followed by Dunnett’s multiple comparisons test), and hA300W did not significantly increase *G*_max_ (P > 0.05 with one-sample *t* test compared with a hypothetical value of 0). In contrast, neither of the site II mutants significantly altered the ability of LIN to shift *V*_50_ (Fig. S9 B).

As a negative control, we introduced similar mutations in the vicinity of the proposed binding residues to test the potential impact of mutating nearby residues predicted to not directly engage with LIN. The hL271A (xL261, near site I) and hI274W (xI264, near site II) mutations did not impair the ability of LIN to shift *V*_50_ (Fig. S9, C–E) or increase *G*_max_ compared with WT (Fig. S9 E, legend). These results support the conclusion that mutation of the predicted hydrophobic binder residues specifically alter LIN–channel interactions. Altogether, these experimental results are in concordance with the prediction from the CG and AA simulations that several hydrophobic residues constitute important binder residues for the PUFA tail in both sites I and II.

### Ability of FA head group to hydrogen bond with S5 tyrosine Y268 is important for LIN/STE selectivity in site I

**Figure 5.**
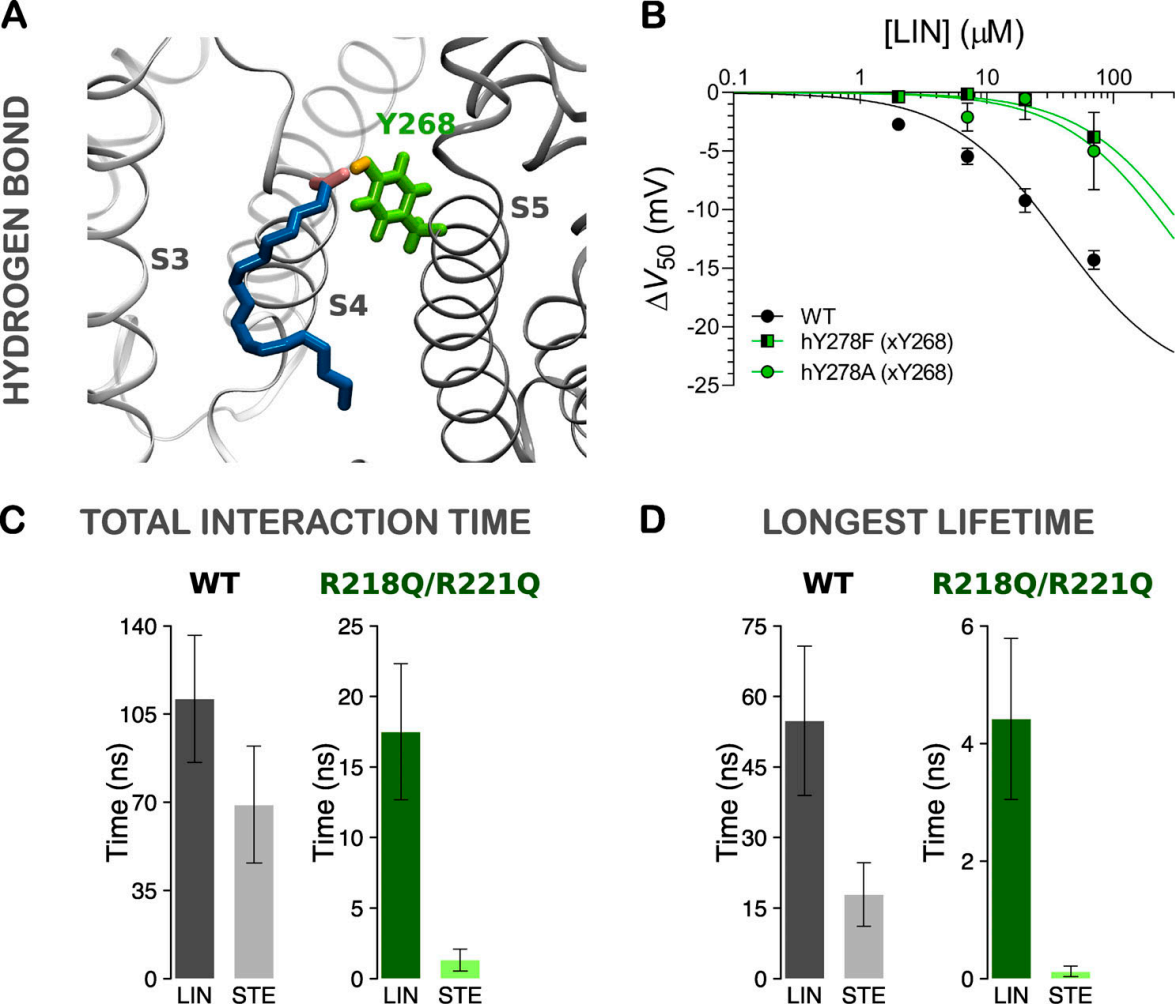
**Hydrogen bonding of LIN and STE head groups to S5 tyrosine comparing the WT (S4 up) and R218Q/R221Q (pseudo S4 down) systems.** **(A)** Side view of a LIN head group interacting with the S5 tyrosine Y268 at site I in the R218Q/R221Q system, which is a pseudo-resting state of S4 generated by mutating in silico the two top arginines in S4. **(B)** Experimental mutation of tyrosine hY278 (xY268) impairs the effect of LIN. Data shown as mean ± SEM; *n* = 4–7 per data point. Concentration-response curves were fitted using Eq. 2 with the Hill coefficient constrained to −1 (see Materials and methods for details). Δ*V*_50,max_ was constrained to −25 mV to make the fits more robust. **(C and D**) Total interaction time (C) and longest lifetime (D) of hydrogen bonds between the FA negatively charged head group and Y268 in 500 ns of AA MD of WT and R218Q/R221Q systems. Data shown as mean ± SEM. Δ*V*_50,max_ denotes maximal effect in Δ*V*_50_.

The predicted interactions between Y268 and the LIN head group through hydrogen bonding (illustrated in Fig. 5 A) suggests that this tyrosine is a novel potential PUFA binding partner. The limited ability of STE to retain close distance to Y268 indicates that the tyrosine may play a role in FA selectivity (Fig. S7 A). We experimentally mutated Y268 to either a phenylalanine (to remove just the OH group) or an alanine to probe the functional impact of this residue. Phenylalanine or alanine mutation of hY278 (xY268) clearly impaired the ability of LIN to shift *V*_50_ (Fig. 5 B), emphasizing the importance of hydrogen-bonding interactions of the amphiphilic side chain of Y268. At 70 µM of LIN, the hY278F and hY278A mutants shifted *V*_50_ by only −5.0 and −3.8 mV compared with −14.3 mV for WT (P < 0.05 with one-way ANOVA followed by Dunnett’s multiple comparisons test to compare with WT). The small remaining effect of LIN on hY278F and hY278A mutants (−5 mV or smaller) is more like the lack of effect of STE at this concentration (0.9 mV) and can be attributed to the residual compensation of the hydrogen-bonding interactions by aromatic or hydrophobic stabilization (Lees-Miller et al., 2009). Thus, experiments support an important role of the S5 tyrosine for LIN effects at site I and suggest that the different ability of LIN and STE to hydrogen bond with Y268 might underlie selectivity at site I.

### Large difference in ability of LIN and STE to interact when outermost S4 arginines are neutralized

Our experimental data show that LIN induces a negative shift in the voltage dependence of activation, suggesting that LIN binds stronger in the activated S4 up state of the voltage sensor than in a resting “S4 down” state of the voltage sensor. In addition, STE does not induce a significant shift in the voltage dependence of activation. To better understand the difference in effect of LIN and STE, we estimated LIN and STE binding to different S4 states. An experimentally determined structure of the S4 down state of KCNQ1 is not yet available. Therefore, we constructed a pseudo S4 down state by neutralizing in silico the two top charges of S4 by introducing the R218Q/R221Q mutations in the model. If S4 moves as in the sliding helix model of S4 (Broomand and Elinder, 2008), where each S4 charge takes the place of its next S4 charge, then, from an electrostatic point of view, removing the two top S4 charges is similar to moving S4 down two “clicks” as a sliding helix (Fig. S10). While this is not a real S4 down state, it provides a simple model to test the importance of R218, R221, and Y268 for PUFA interaction with KCNQ1. We positioned either LIN or STE in each of the channel’s site I (i.e., four sites per channel) according to representative binding poses identified in the AA simulations and performed similar simulations for the R218Q/R221Q system as for the WT (S4 up) system. Three replicates of each system were simulated for 500 ns each (providing simulation data for a total of 12 LIN or STE molecules, respectively, in site I). In the WT system, there is a 2–3-fold difference in ability of LIN and STE to hydrogen bond with Y268 (Fig. 5, C and D; and Fig. S10). Neutralizing R218 and R221 reduces LIN’s ability to hydrogen bond with Y268, which is observed as reduced total interaction time and longest lifetime of roughly a factor of 6–12 in the R218Q/R221Q system compared with the WT system (Fig. 5, C and D; and Fig. S10). This likely reflects decreased stabilization of LIN in site I upon removal of the electrostatic coordination of LIN head group by S4 arginines. In the R218Q/R221Q system, there is a dramatic difference in the ability of LIN compared with STE to hydrogen bond with Y268: there are 13- and 37-fold differences in total interaction time and longest lifetime, respectively, for LIN compared with STE (Fig. 5, C and D; and Fig. S10). This suggests that once the electrostatic coordination of FA head group by S4 arginines is lost (as in the predicted down state of S4), STE largely fails to hydrogen bond with Y268.

We extended the simulation of one LIN and one STE R218Q/R221Q system to 5 µs to analyze occupancies of LIN and STE head groups at site I across the membrane normal with 0.5-Å slabs averaging for each consecutive 1-µs interval. These simulations revealed a 20–300-fold difference in occupancy of LIN compared with STE within 3 Å of site I (Fig. S11 A). The differences in interaction of LIN and STE in the R218Q/R221Q systems are further illustrated by occupancies of head groups around R218 and Y268. LIN displays an 8–30-fold difference compared with STE in occupancy within 3 Å of R218 (Fig. S11 B) and 60–300-fold difference in head group occupancy within 3 Å of Y268 (Fig. S11 C). Altogether, these data suggest that LIN binds better to KCNQ1 in the S4 up state than in the S4 down state and that LIN binds better than STE in both states with the largest difference observed for the S4 down state. These data also suggest that Y268 is a critical anchor point for the FA head group and an important determinant of FA selectivity at site I.

## Discussion

Our results here clearly suggest that there are two functionally important PUFA binding sites present in KCNQ1, one close to the voltage sensor and one close to the pore. In both regions, the negatively charged head group of the PUFA has long-lived and frequent contacts with a positively charged KCNQ1 residue, R218 in the voltage sensor and K316 in the pore domain. We discovered a novel head group interaction—a hydrogen bond between Y268 and the negative PUFA head group—with KCNQ1 at the voltage sensor site. We also discovered several hydrophobic interactions between the PUFA tail and KCNQ1 residues at both sites. The hydrophobic tail of the PUFA is more mobile and samples contacts with many hydrophobic KCNQ1 residues during one interaction event of the negatively charged PUFA head group with the positively charged KCNQ1 residue.

We propose that it is mainly an electrostatic interaction between the negative PUFA head group and the top positive S4 charges that causes the shift in voltage dependence of activation. The electrostatic force between PUFA and R218 in the down state will attract S4 from the down state to the up state, thereby speeding up activation of the channel. Similarly, the electrostatic force between PUFA and R218 in the up state will try to retain S4 from leaving the up state and go to the down state, thereby slowing deactivation of the channel. In experiments, we observed LIN-induced acceleration of KCNQ1 opening kinetics and slowing down of closing kinetics (Fig. S12). These two effects, speeding up of activation and slowing down of deactivation, will result in a shift in the voltage dependence of activation. We propose that Y268 functions as an anchor point for the PUFA head group, thereby allowing a more immobile PUFA head group to attract the mobile S4 charges toward it. For these effects to work, the PUFAs do not have to be bound to the channel sites at all times. The measurable effects on macroscopic parameters are due to time averaging of the PUFA-induced changes in the probabilities of transitions between conformational states. Even if the PUFAs are not constantly bound to the channel, they will still affect the macroscopic parameters by increasing or decreasing the probability of transitions during the times they are bound. Better binding of PUFA to the S4 up state than to the S4 down state is consistent with PUFA stabilization of the activated S4.

What could be a possible molecular mechanism explaining PUFA impact on the permeation properties of KCNQ1? We previously proposed that the PUFA effect on permeation is due to conformational changes in the pore due to electrostatic interaction between the PUFA head group and K316 in S6 (Liin et al., 2018). These conformational changes are unlikely to be accurately sampled in an equilibrium MD simulation. In a previous simulation study (Liin et al., 2018), we instead used a strong force on K316 in the direction of the putative PUFA localization in the lipid bilayer to speed up these conformational changes. We found that this force induced conformational changes in the pore that would explain an increased K^+^ conduction observed experimentally. PUFA binding to site II identified in this study places the PUFA head group in a position that is expected to cause electrostatic pulling on K316 in a direction aligning with the previous pulling experiments (Liin et al., 2018). The role of each site for distinct PUFA effects is further supported by our finding that mutation of residues predicted to constitute site I predominantly affect the ability of LIN to shift *V*_50_, whereas mutation of residues predicted to constitute site II predominantly affect the ability of LIN to increase *G*_max_. It should, however, be noted that the effect on *G*_max_ may be influenced also by other factors, such as the intrinsic open probability of a mutant (Liin et al., 2018).

In comparison to the PUFA LIN, we find that the saturated STE interacts much less with KCNQ1. This is most likely due to the more rigid nature of the saturated alkane chain of STE. The saturated tail of STE does not explore as many different conformations in a short time period as the polyunsaturated tail of LIN and therefore does not explore as many stabilizing interactions with KCNQ1 residues as shown here for LIN with KCNQ1 residues. For site II, there are clearly fewer interactions for STE compared with LIN in all simulations. For site I, the apparent difference in protein–lipid interactions between STE and LIN is illustrated by the simulations of the R218Q/R221Q system (pseudo S4 down state). STE interactions with site I are 10 and 40 times less likely and long lasting, respectively, than for LIN. The impairment of STE interactions causes large differences in LIN/STE occupancy at site I in the R218Q/R221Q system. The observed reduction in STE–KCNQ1 interactions in the R218Q/R221Q system may explain the lack of effect of STE on the activation time course in KCNQ1. The mobility enabled by the level of unsaturation in the alkyl tail has previously been shown through NMR studies, where the DHA has been observed to exhibit rapid conformational transitions with short correlation times as well as exceptionally low-order parameters (Eldho et al., 2003; Soubias and Gawrisch, 2007). An enhanced mobility of alkyl tails in membrane environment has also been demonstrated by ab initio calculations in combination with AA MD simulations of DHA. The presence of double bonds in the lipid tails leads to low torsional energy barriers for the rotatable bonds in the DHA chain and explains the differences in mobility between polyunsaturated and saturated lipid chains (Feller, 2008). The differences in LIN versus STE tail interactions with a hydrophobic-aromatic cassette in sites present in KCNQ1 is in general agreement with the experimental and modeling results of Ballweg et al. (2020) and Palermo et al. (2015). The previous studies and our results emphasize the critical importance of chain unsaturation, which impacts mobility of the tail and hence an entropic component of binding.

What could be expected for other types of FAs? Monounsaturated FAs have a tail with mobility properties more similar to those of saturated tails than PUFAs have (Palermo et al., 2015; Leng et al., 2018). We therefore expect that monounsaturated FAs would bind poorly to sites I and II, similarly to STE as shown here. This is in agreement with previous experimental data showing a lack of activating effect of the monounsaturated FA oleic acid on KCNQ1 (Liin et al., 2015). In contrast, we expect that other PUFAs such as DHA or eicosapentaenoic acid or PUFA analogues such as N-AT would bind well overall to these sites, similarly to LIN in this study, due to the more mobile tail. However, there might be some differences in how PUFA interacts with KCNQ1, depending on the spatial location of the double bonds in the PUFA tails, which may play a role in specific interactions with aromatic moieties in proteins (Palermo et al., 2015). This is in agreement with previous experimental data showing activating effects on KCNQ1 of a large set of PUFAs (including DHA, eicosapentaenoic acid, and N-AT) but with some variability depending on the location of double bonds (Bohannon et al., 2019). When assessing the importance of Y268 for the *V*_50_ effect of the PUFA DHA and PUFA analogue N-AT on KCNQ1, we find it to be critical for the DHA effect (Fig. S13; DHA has no effect on hY278F) but not for the N-AT effect (Fig. S13; N-AT has preserved effect on hY278F). This suggests that for PUFAs with carboxyl head groups, Y268 is an important anchor point, while PUFA analogues with other head groups might interact with the channel in a slightly different way. Future studies are needed to resolve the atomistic details of how double bond location in the PUFA tail affects PUFA interactions with the aromatic cassette and how PUFA analogues with different head groups interact with KCNQ1.

Many different mechanisms have been proposed to explain the effects of PUFA on different ion channels. Indirect effects, such as altering the membrane fluidity or affecting second messenger pathways, have been proposed to explain the relative broad selectivity of PUFAs (Boland and Drzewiecki, 2008; Elinder and Liin, 2017). Moreover, different effects of polyunsaturated and saturated FAs on mechanical properties of the membrane may underlie ion channel effects, but also differences in their abilities to make specific interactions with aromatic residues (Lundbæk et al., 2010; Romero et al., 2019; Ballweg et al., 2020). Although we cannot rule out that PUFAs have indirect effects on KCNQ1 function, our results here are consistent with direct PUFA effects on KCNQ1 by binding to two separate sites on KCNQ1. Moreover, our previous experimental data showing that altering the charge of the PUFA head group without altering the PUFA tail or mutating specific residues in KCNQ1 changes the effect of PUFA on KCNQ1 channels (Liin et al., 2015; Larsson et al., 2018) are more consistent with a direct electrostatic interaction between the head group and KCNQ1 residues. Direct PUFA effects on ion channels were proposed earlier. For instance, electrostatic interactions are important for PUFA effects on the Shaker K^+^ channel (Börjesson et al., 2008; Ottosson et al., 2014; Yazdi et al., 2016), whereas an aromatic interaction is important for PUFA effects on the BK channel (Hoshi et al., 2013; Tian et al., 2016).

However, for many ion channels, the mechanistic basis for direct interaction remains unknown. We anticipate that the approach of combined CG and AA simulations used in this study, which allows an unbiased exploration of interaction across longer timescales as well as capturing finer atomistic details of interaction, will aid in resolving lipid interaction with the diverse ion channels, whose function has been shown experimentally to be modulated by FAs. Altogether, the characterization of the interactions between PUFAs and KCNQ1 performed in this study enables mechanistic understanding of PUFA-mediated activation of KCNQ1. These insights may be used in future development of drugs based on PUFA mechanisms. For instance, KCNQ1 together with its β subunit KCNE1 form voltage-gated potassium channels that generate the important repolarizing I_Ks_ current in the heart (Barhanin et al., 1996; Sanguinetti et al., 1996; Nerbonne and Kass, 2005). Because loss-of-function mutations in KCNQ1 or KCNE1 predispose the heart to arrhythmia, development of KCNQ1/KCNE1 channel activators using PUFA binding sites and similar underlying electrostatic mechanisms may be an anti-arrhythmic strategy. However, many important questions remain to be addressed, such as how KCNE1 impacts PUFA binding to KCNQ1 and how PUFAs target other cardiac ion channels.

## Supplementary Material

Table S1 summarizes the biophysical properties of KCNQ1 mutants.
