# Supplementary material for: Identification of PUFA interaction sites on the cardiac potassium channel KCNQ1
Source: J Gen Physiol. 2021 May 3;153(6):e202012850. doi: 10.1085/jgp.202012850 (PMC8097404; doi:10.1085/jgp.202012850)
Supplement: Table S1 — summarizes the biophysical properties of KCNQ1 mutants. [file JGP_202012850_TableS1.docx]

| **Table SI. Summary of biophysical properties of used constructs.** | | | |
| --- | --- | --- | --- |
| **Construct** | ***V*_50_**  **(mV)** | ***s***  **(mV)** | ***n*** |
| hKCNQ1 WT | −26.2 ± 0.9 | 9.7 ± 0.2 | 14 |
| hKCNQ1_F232A | −12.2 ± 2.6 | 9.4 ± 0.5 | 7 |
| hKCNQ1_R249Q | −13.7 ± 0.7 | 10.2 ± 0.4 | 5 |
| hKCNQ1_R259Q | −9.9 ± 1.4 | 11.1 ± 0.4 | 5 |
| hKCNQ1_L271A | −10.3 ± 1.3 | 18.2 ± 0.4 | 5 |
| hKCNQ1_L273W | −15.8 ± 1.3 | 12.4 ± 0.8 | 6 |
| hKCNQ1_I274W | −33.4 ± 2.3 | 11.8 ± 0.3 | 5 |
| hKCNQ1_Y278A | −13.1 ± 5.7 | 14.2 ± 1.2 | 8 |
| hKCNQ1_Y278F | −22.0 ± 1.7 | 9.8 ± 0.2 | 5 |
| hKCNQ1_F279A | −29.2 ± 0.5 | 7.6 ± 0.1 | 10 |
| hKCNQ1_L282A | +1.2 ± 1.8 | 12.9 ± 1.0 | 4 |
| hKCNQ1_A300W | −11.0 ± 1.7 | 9.9 ± 0.6 | 5 |
| hKCNQ1_L303W | n.e. |  |  |
| hKCNQ1_L303F | n.e. |  |  |
| hKCNQ1_V307W | n.e. |  |  |
| hKCNQ1_V307F | n.d. |  |  |
| hKCNQ1_S330W | n.e. |  |  |
| hKCNQ1_S330F | n.e. |  |  |
| Data shown as mean ± SEM. *V*_50_ is the midpoint of the *G*(*V*) curve and *s* the slope factor in control solution, estimated from the Boltzmann fits (see Materials and Methods for details), and *n* the number of recordings. n.e. denotes no expression. n.d. denotes not determine (because of inconsistent channel behavior). | | | |
